# Brn3a regulates neuronal subtype specification in the trigeminal ganglion by promoting Runx expression during sensory differentiation

**DOI:** 10.1186/1749-8104-5-3

**Published:** 2010-01-22

**Authors:** Iain M Dykes, Jason Lanier, S Raisa Eng, Eric E Turner

**Affiliations:** 1 Department of Psychiatry, University of California, San Diego, 9500 Gilman Drive, La Jolla, CA 92093-0603, USA; 2 VA San Diego Healthcare System, San Diego, CA, USA; 3 Seattle Children's Research Institute, 1900 9th Avenue, Seattle, WA, 98101, USA

## Abstract

The transcription factor Brn3a, product of the *pou4f1 *gene, is expressed in most sensory neurons throughout embryogenesis. Prior work has demonstrated a role for Brn3a in the repression of early neurogenic genes; here we describe a second major role for Brn3a in the specification of sensory subtypes in the trigeminal ganglion (TG). Sensory neurons initially co-express multiple Trk-family neurotrophin receptors, but are later marked by the unique expression of TrkA, TrkB or TrkC. Maturation of these sensory subtypes is known to depend on the expression of Runx transcription factors. Newborn Brn3a knockout mice fail to express TrkC, which is associated in the TG with mechanoreceptors, plus a set of functional genes associated with nociceptor subtypes. In embryonic Brn3a^-/- ^ganglia, the normal expression of Runx3 is never initiated in TrkC^+ ^neurons, and Runx1 expression is greatly attenuated in TrkA^+ ^nociceptors. These changes are accompanied by expanded expression of TrkB in neurons that abnormally express multiple Trks, followed by the loss of TrkC and TrkA expression. In transgenic embryos expressing a Brn3a-VP16 dominant transactivator, Runx3 mRNA expression is increased, suggesting that it is a direct regulatory target of Brn3a. Chromatin immunoprecipitation confirms that Brn3a binds *in vivo *to a conserved upstream enhancer element within histone H3-acetylated chromatin in the *Runx3 *locus. Together these data show that Brn3a acts upstream of the Runx factors, which then repress TrkB expression to allow establishment of the non-overlapping Trk receptor profiles and correct terminally differentiated phenotypes.

## Background

Sensory neurons of the dorsal root ganglia (DRG) and trigeminal ganglia (TG) convey somatosensory information to the spinal cord and brainstem. During embryonic development, sensory neurons differentiate into three primary subtypes: nociceptors (pain), mechanoreceptors (touch), and proprioceptors (muscle tension). In the DRG, these are characterized by the expression of the neurotrophin receptors TrkA, TrkB and TrkC, respectively. In the TG, proprioceptors for the muscles of mastication reside in the mesencephalic trigeminal (mesV) within the central nervous system, and TrkC is expressed in subsets of mechanoreceptors [1]. In perinatal development, nociceptors further differentiate into peptidergic and non-peptidergic phenotypes, the latter being distinguished by downregulation of TrkA and expression of Ret [2,3].

Sensory neurogenesis is dependent on the expression of the basic helix-loop-helix (bHLH) transcription factors Neurog1 and Neurog2 [4,5]. Mice lacking Neurog2 fail to generate early-born DRG proprioceptors, an effect that is later compensated by Neurog1, while mice lacking both Neurog factors show global failure in sensory neurogenesis [6]. The subsequent specification of DRG proprioceptors and nociceptors is dependent on the runt-domain transcription factors Runx3 and Runx1, which maintain and refine the expression of Trk receptors, and lead to the expression of subtype-specific functional genes and correct innervation of central nervous system targets [7,8].

Beginning just prior to cell cycle exit, nearly all embryonic sensory neurons co-express the POU-homeodomain transcription factor Brn3a and the LIM-homeodomain factor Islet1 [9]. Sensory neurons lacking these factors exhibit multiple defects in sensory axon growth, and die in the perinatal period [10-12]. In the DRG and TG, Brn3a facilitates the progression of sensory development by terminating the expression of neurogenic bHLH factors by direct repression [13,14], and a similar role has been described for Islet1 [15], defining one common function for these pan-sensory factors.

It is less clear what role Brn3a plays in defining sensory subtypes. Although pan-sensory transcription factors are not obvious candidates for subtype specification, previous studies have shown disproportionate loss of some subtype markers in the Brn3a knockout [16-18], and Islet1 knockout DRG exhibit a loss of nearly all nociceptors in the developing ganglia, while TrkC^+ ^proprioceptors are relatively spared [15].

Here we demonstrate a role for Brn3a in subtype specification during TG development. At birth, the TG of Brn3a^-/- ^mice exhibit a profound loss of TrkC, Runx3, and multiple nociceptor channels and receptors, while sensory neuropeptide expression is maintained. During embryogenesis, Brn3a is required to initiate normal expression of the fate selector genes *Runx3 *and *Runx1*. At midgestation, Brn3a^-/- ^TG show expanded TrkB expression compared to wild type, resulting in failure to segregate TrkB^+ ^from TrkC^+ ^and TrkA^+ ^neurons, effects that are consistent with the loss of Runx function. Chromatin immunoprecipitation demonstrates that Brn3a interacts with a *Runx3 *upstream enhancer *in vivo*, revealing the first direct positively regulated Brn3a target. Together these data show that Brn3a creates a permissive condition for the expression of the Runx factors, which then repress TrkB expression and allow differentiation of the principal sensory subtypes.

## Methods

### Animals and matings

Mice carrying a *Brn3a *null allele [10] or a *TauLacZ *transgene replacing the *Brn3a *coding sequence [19] have been previously described, and mice heterozygous for these alleles were maintained in a C57bl/6 background. For timed matings, noon of the day on which mucous plugs were identified was designated embryonic day 0.5 (E0.5). The age of harvested embryos was confirmed using morphological criteria and body length [20]. Embryos expressing a Brn3a-VP16 dominant transactivator in sensory ganglia were generated by transgenic expression of a construct containing a VP16 activation domain fused to sequences encoding the carboxy-terminal 238 amino acids of Brn3a, including the POU-specific and POU-homeodomains. Brn3a-VP16 was expressed under control of an 11-kb *Brn3a *sensory enhancer/promoter, in which the native sequences mediating Brn3a binding have been mutated to eliminate negative autoregulation [21].

### Immunofluorescence and *in situ *hybridization

Embryos for immunofluorescence and *in situ *hybridization studies were generated by timed matings as described above. Embryos were fixed by immersion in 4% paraformaldehyde for 30 minutes (E10.5) or 2 hours (E13.5). Newborn pups were fixed by transcardiac perfusion with 4% paraformaldehyde; the cranium was then opened and the brain was post-fixed 2 hours in the same fixative. Tissue was embedded in Neg50 (Thermo Fisher, Waltham, MA, USA) and sectioned on a cryostat. Slides were incubated overnight at 4°C in primary antisera, washed then incubated with Alexa 488 or 594 dye-conjugated secondary antibodies (Invitrogen, Carlsbad CA, USA) for 1 hour at room temperature and mounted in Vectashield (Vector Laboratories, Burlingame, CA, USA). Polyclonal rabbit antiserum against Brn3a has been previously described [22]. Other antisera used included rabbit antisera against Etv1/Er81, Runx1 and Runx3 obtained from Dr Sylvia Arber [23,24], rabbit antisera against TrkA obtained from Dr Louis Reichardt, and guinea pig antisera against Islet2 obtained from Dr Sam Pfaff [25]. Immunofluorescence for other antigens was performed with commercially available antibodies, including goat antisera against TrkB and TrkC (R&D Systems, Minneapolis, MN, USA), rabbit antiserum against TrkB (Cell Signaling, Danvers, MA, USA), goat antiserum against cRET (Fitzgerald, Acton, MA, USA) goat antiserum against LacZ (Biogenesis/MorphoSys, Kingson, NH, USA) and rabbit antiserum against Islet1 (Abcam, Cambridge, MA, USA).

*In situ *hybridization was performed on cryostat sections using digoxigenin labeled cRNA probes as previously described [26]. *In situ *hybridization probes for peptidergic and non-peptidergic sensory markers were gifts of Qiufu Ma and have been previously described [7,27]. A cDNA encoding Htr3a was a gift of Allan Basbaum [28].

### Luciferase transfection assays

Transient transfections for luciferase activity assays were performed in CV-1 epithelial cells by lipofection. Luciferase reporter constructs for transfection assays contained three copies of a Brn3a recognition site linked to a minimal promoter derived from the rat prolactin gene in the vector pGL-2 [29]. The oligonucleotide sequences used to make the reporter construct for the consensus Brn3a recognition element were GATCTCTCCTGC*ATAATTAAT*TACGCCCG and GATCCGGGCGTA*ATTAATTAT*GCAGGAGA (core binding site in italics). An effector plasmid consisting of a VP16 activation domain fused to Brn3a, identical to that used for the transgenic misexpression of VP16-Brn3a, was constructed in the expression vector pcDNA1-amp. Luciferase assays were performed as previously described [30].

### Microarray analysis

For the generation of Brn3a^+/+ ^and Brn3a^-/- ^TG samples, Brn3a heterozygote mice were mated and the embryos were harvested at E13.5. Transgenic founder animals carrying the Brn3a-VP16 construct were also dissected at E13.5. TG were removed by blunt dissection and carefully freed of adherent non-neural tissue with fine forceps. Dissected ganglia were placed in RNAse inhibitor solution (RNAlater, Ambion, Austin, TX, USA), and RNA was prepared using the RNeasy system (Qiagen, Valencia, CA, USA). Embryos were genotyped for *Brn3a *alleles using quantitative PCR from a sample of tail or hind-limb tissue harvested at the time of ganglion dissection. For analysis of *Brn3a *knockout and control ganglia, TG from five embryos were pooled for a single microarray analysis. For analysis of VP16-Brn3a transgenic ganglia, RNA was prepared from a pair of TG from a single E13.5 embryo, yielding 0.5 to 1.0 μg of RNA. Three VP16-Brn3a and three littermate control samples were harvested, allowing a total of nine semi-independent comparisons between VP16 and control samples. The VP16-Brn3a microarray results were then compared to the ranked lists of transcripts increased and decreased in the E13.5 Brn3a^-/- ^ganglia to identify a subset of these changes that may indicate direct targets (Additional file 1). The microarray analyses of Brn3a^-/- ^and control ganglia used for comparison purposes appear in detail elsewhere [31].

The generation of cDNA, production of labeled cRNA with a single T7 amplification step, and hybridization to GeneChip arrays were all performed according to standard protocols provided by the manufacturer (Affymetrix, Santa Clara, CA, USA). Gene expression analysis was performed using the Affymetrix 430v2 array. The primary analysis of microarray data, including determination of the absence/presence of the assayed transcripts, transcript expression levels, and the probability of change in transcript expression between samples ('change *P*') was performed with Microarray Suite 5.0 (MAS5, Affymetrix). Default MAS5 parameters were used for increase (I) and decrease (D) calls, which were *P *< 0.002 and *P *> 0.998 for I and D, respectively. All array values were scaled to a target value of 500 using global scaling. Microarray probe sets were related to the corresponding mouse transcripts using the NetAffx database (Affymetrix), based on the NCBI Build 36 annotation of the mouse genome.

### Electrophoretic mobility gel shift assays

Recombinant Brn3a protein was produced by transfecting HEK293 cells with a plasmid containing the full coding sequence of Brn3a in the vector pcDNA1-amp using Lipofectamine 2000 (Invitrogen). Cells were suspended in a high-salt extraction buffer containing 20 mM HEPES pH7.9, 25% glycerol, 450 mM NaCl and 0.2 mM EDTA. Protease inhibitors were added from the Complete Mini tablet (Roche Applied Science, Indianapolis, IN, USA) and cells lysed by sonication. Cellular debris was removed by centrifugation and the resulting supernatant protein extract was collected.

Synthetic double-stranded oligonucleotide probes that varied in length from 39 to 79 bp were end-labeled with γ^32^P by polynucleotide kinase (New England Biolabs, Beverly, MA, USA). Brn3a protein HEK cell lysate or control untransfected cell lysate was incubated with the probe for 20 minutes in a binding buffer consisting of 20 mM Tris, pH 8.0, 100 mM KCl, 5 mM MgCl_2 _, 0.2 mM EDTA, 100 mg/ml poly(dI-dC), 100 mg/ml bovine serum albumin, 10% glycerol and 1 mM DTT. Polyclonal rabbit anti-Brn3a antiserum was added to some samples prior to gel loading to obtain an antibody supershifted band. Electrophoresis was performed on a 5% polyacrylamide gel (Biorad, Hercules, CA, USA). The sequences of all oligonucleotide probes used in the electrophoretic mobility gel shift assay (EMSA) are listed in Additional file 2.

### Chromatin immunoprecipitation

The consensus binding site of Brn3a and conserved variants of this sequence that permit high affinity binding have been previously determined [29]. Candidate Brn3a binding sites corresponding to these sequences in the *Runx3 *locus and other potential target gene loci were determined using the search functions of Lasergene (DNAstar, Madison, WI, USA). Cross-species sequence conservation within the Runx3 locus was analyzed using the UCSC genome browser.

Methods for chromatin immunoprecipitation (ChIP) have been previously published [14]. Briefly, trigeminal ganglia were dissected from E13.5 wild-type ICR mouse embryos. Proteins were fixed to DNA by formaldehyde treatment and DNA was sonicated to an average size of 500 bp. Selection was performed using either a polyclonal rabbit antibody to Brn3a [22] or a polyclonal rabbit antibody to acetylated histone H3 (Upstate Biotechnology/Millipore, Billerica, MA, USA) bound to an anti-rabbit IgG magnetic bead (Invitrogen, Carlsbad CA, USA). Two or three quantitative PCR primers encompassing approximately 70-bp amplicons were designed to sequences close to each of the identified potential binding sites. Quantitative PCR was performed using an Applied Biosystems (Foster City, CA, USA) 7300 thermocycler and enrichment of the selected chromatin relative to an unselected control sample was analyzed by the cycle threshold difference method [32]. A baseline (one-fold enrichment) for data normalization was set using control primers against a region of the *Alb *(serum albumin) locus that is not transcribed in sensory neurons and does not contain Brn3a binding sites [14]. The sequences of all primers used in the ChIP assay are listed in Additional file 3.

## Results

### Brn3a expression is maintained in all sensory subtypes throughout embryogenesis

**Figure 1:**
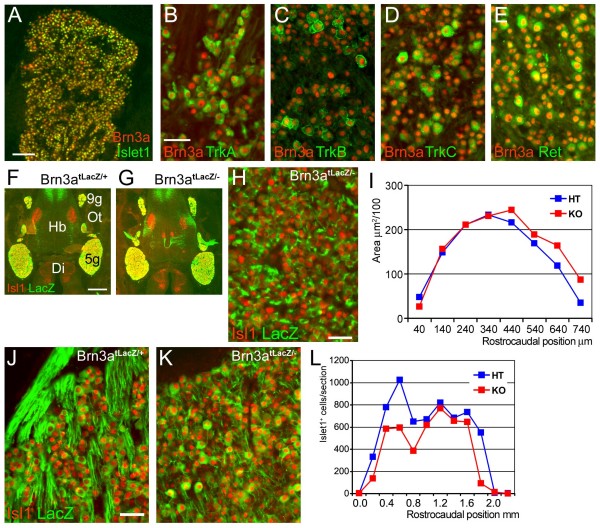
**Pan-sensory expression of Brn3a and survival of a majority of trigeminal neurons in Brn3a knockout mice**. **(A-E) **Co-localization of Brn3a with specific sensory markers in the TG in transverse sections of P0 wild-type ganglia. Brn3a is co-localized with the pan-sensory marker Islet1 and the subtype-specific markers TrkA, TrkB, TrkC, and Ret. **(F-I) **Islet1 expression and LacZ expressed from the Brn3a locus (*Brn3a*^*tLacZ *^allele) were used to assess TG morphology and marker expression at E13.5. Ganglion morphology is grossly normal (F, G), and Islet1 continues to be expressed in LacZ^+ ^neurons in Brn3a knockout embryos (H). No significant difference in the volumes of Brn3a^tLacZ/- ^and Brn3a^tLacZ/+ ^control TG was observed at this stage (I). The zero coordinate is the rostral pole of the ganglion. Paired T test for comparison of coronal section areas across the rostrocaudal extent of the ganglion: n = 8, *P *= 0.12, not significant. **(J-L) **At P0.5 Islet1 expression continued to identify all or nearly all LacZ^+ ^neurons in Brn3a^tLacZ/- ^knockout and Brn3a^tLacZ/+ ^control TG (J, K, transverse section). Islet1 immunoreactive nuclei were counted in serial sections across the rostrocaudal extent of the TG (L), and mean cell number was reduced from 696 to 501 nuclei per section (72% remaining), with cell loss concentrated in the rostral pole of the ganglion. Paired T-test for comparison cell number in matched coronal sections across the entire rostrocaudal extent of the ganglion: n = 12, *P *= 0.01. 5 g, trigeminal ganglion; 9 g, geniculate ganglion; Di, diencephalon; Hb, hindbrain; HT, heterozygote control; KO, knockout; Ot, otic region.

In prior studies we have shown that Brn3a is expressed in all or nearly all trigeminal precursors at midgestation [9,26]. To determine whether Brn3a expression is maintained in all of the principal neuronal subtypes in the TG, we examined the TG of pups at postnatal day 0.5 (P0), the last day on which Brn3a knockout mice are viable. We found that Brn3a is co-expressed with the pan-sensory transcription factor Islet1 at this stage, although the relative signals for the two proteins vary (Figure 1A). Brn3a was also co-expressed with markers for the principal sensory subtypes, including the nerve growth factor receptor TrkA (Figure 1B), the brain-derived neurotrophic factor and neurotrophin-4 receptor TrkB (Figure 1C), the neurotrophin-3 receptor TrkC (Figure 1D) and the glial cell line-derived neurotrophic factor receptor RET (Figure 1E). Thus, it is plausible that Brn3a may play a role in the development of multiple subtypes of trigeminal neurons.

### Cell death is limited in the Brn3a knockout

Prior studies have reported significant cell loss in the sensory ganglia of Brn3a knockout mice. Huang *et al*. [18], using Nissl staining, reported that 29% of total TG neurons remained at P0. Lei *et al*. [33], using the neuronal marker NeuN, reported 31% of neurons remaining, whereas Xiang *et al*. [10] reported reduction of the cross-sectional area of the Brn3a^-/- ^TG to 56% of controls. In order to clearly distinguish changes in cell fate from the complete absence of a significant fraction of sensory neurons, we first determined whether the Brn3a^-/- ^TG was normal in size and cellularity in our samples at E13.5 and P0.5. Islet1 was employed as a specific nuclear marker for sensory neurons because its expression does not change in Brn3a knockout ganglia. At E13.5 there was no apparent difference between the size of the TG in Brn3a^tLacZ/+ ^and Brn3a^tLacZ/- ^embryos (Figure 1F, G). Immunofluorescence for Islet1 showed clear nuclear outlines in Brn3a^tLacZ/- ^ganglia (Figure 1H), and measurement of the cross-sectional area of the trigeminal at E13.5 showed no significant difference between heterozygous and knockout embryos (Figure 1I), consistent with prior studies [12,18]. At P0, Islet1 staining and nuclear morphology remained normal, although there was a marked disturbance in the appearance of labeled axonal fibers within the ganglion (Figure 1J, K). Compared to prior studies, we observed a more modest loss of TG neurons, concentrated in the rostral pole of the ganglion, and we found that approximately 72% of the normal complement of TG neurons were present at birth in the Brn3a knockout (Figure 1L). Potential reasons for the differing results appear in the Discussion.

### TrkC, Runx3 and nociceptor markers are selectively lost in the Brn3a knockout

**Figure 2:**
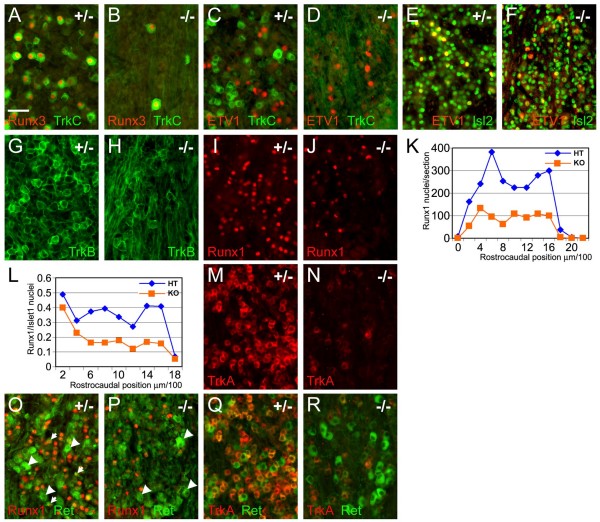
**Loss of Brn3a profoundly affects the major sensory subtypes at birth**. Expression of markers of known sensory subtypes were examined at P0 in transverse sections of Brn3a knockout and heterozygote control TG. Heterozygote controls are phenotypically normal and have been previously shown to have minimal changes in global gene expression [26]. **(A-F) **Expression of TrkC, Runx3, and Etv1. TrkC and Runx3 immunoreactive neurons are reduced to less than 10% of controls. Small Etv1 immunoreactive neurons, most of which co-express Islet2, are unaffected. **(G, H) **Neurons expressing TrkB persist in Brn3a^-/- ^mice at P0, but the distribution of the protein is markedly altered, with increased expression in axons and less expression in discrete cell bodies. **(I-R) **Expression of nociceptor markers Runx1, TrkA and Ret. The absolute number of Runx1 positive neurons is significantly decreased across the rostrocaudal extent of the ganglion (K, paired *t*-test, *P *= 0.001), and this loss is in excess of the overall decrease in cell number as assayed by Islet1 expression (L, paired *t*-test, *P *= 0.0004). TrkA expression is markedly diminished in the knockout (M, N). Small diameter neurons co-expressing Runx1 and Ret (O, arrows) are absent from the knockout, but a population of large diameter Ret^+ ^neurons that are negative for Runx1 and TrkA, and may represent a class of mechanoreceptors, persist (O, P, arrowheads). HT, heterozygote control; KO, knockout.

We next examined markers of sensory subtypes in the TG of Brn3a knockout mice at P0, the last stage that could be examined owing to the neonatal death of the mutants (Figure 2). In the DRG, proprioceptors co-express TrkC and Runx3 [34], and Ia somatic muscle afferents also express ETV1 [35], but this population appears to be absent from the trigeminal [13] because the corresponding proprioceptors innervating the muscles of mastication are located in the mesencephalic trigeminal (mesV). In the TG, at least some of the TrkC-expressing neurons are mechanoreceptors [1]. As expected, TrkC and Runx3 were co-expressed in a population of large diameter cells in the wild-type ganglion (Figure 2A). Consistent with prior observations [11,18], we observed an almost complete loss of TrkC in the Brn3a knockout, and Runx3 expression was also nearly absent (Figure 2B). ETV1 was observed in a population of small diameter cells in the trigeminal. These cells were negative for TrkC (Figure 2C) but co-expressed Islet2 (Figure 2E), which together with their small size suggests that they are not proprioceptors. ETV1 expression in these neurons was largely unaffected by loss of Brn3a (Figure 2D, F).

In the wild-type ganglion we observed expression of TrkB in a population of large diameter cells (Figure 2G). Relatively little is known about the sensory modalities characterized by TrkB expression, but these cells are likely to include mechanoreceptors such as Ruffini afferents and Pacinian corpuscles [36,37]. Expression of TrkB was maintained in the knockout, but with a marked shift in the localization of the antigen from the cell body to axons within the ganglion (Figure 2H). The persistence of detectable TrkB expression at P0 was confirmed by *in situ *hybridization (Additional file 4). Thus, TrkB mechanoreceptors appear to be specified in the Brn3a knockout, but their development is abnormal.

Prior work has shown that nociceptors are identified by expression of several markers in a complex and changing pattern. Early in development all nociceptors co-express TrkA and Runx1, while in postnatal development the non-peptidergic subset of nociceptors downregulate TrkA and begin to express Ret along with Runx1. Peptidergic neurons maintain expression of TrkA, lose Runx1 expression, and never express Ret [2,7,38]. Due to perinatal lethality, only the initial part of this process could be examined in Brn3a knockouts.

In the Brn3a knockout TG we observed a marked reduction in the level of Runx1 expression (Figure 2I, J), and the number of cells positive for Runx1 was reduced by an average of 64% across the extent of the ganglion (Figure 2K). Correction for cell loss using Islet1 to identify surviving neurons within the ganglion showed that the proportion of cells in the ganglion positive for Runx1 was reduced from 34 ± 3% to 18 ± 3% (Figure 2L), demonstrating a specific reduction in Runx1 expression in the absence of Brn3a.

The TG of newborn Brn3a^-/- ^mice also exhibited changes in the expression of the sensory subtype markers TrkA and Ret. The number of TrkA^+ ^neurons was markedly reduced at P0 (Figure 2M, N), consistent with previous reports [18,39]. In contrast, abundant Ret^+ ^neurons persisted in the Brn3a^-/- ^TG (Figure 2P, R). Ret is expressed in non-peptidergic nociceptors, which initially co-express TrkA [38], and also in earlier-developing neurons with large cell bodies, which are likely to include certain classes of mechanoreceptors [40]. In the Brn3a^-/- ^TG at P0 the persisting Ret^+ ^neurons appear to belong to the latter class, in that they have large cell bodies, and do not co-express Runx1 (Figure 2O, P), or TrkA (Figure 2Q, R). Thus, although the large Ret^+^/Runx1^- ^neurons express Brn3a (Figure 1E), they do not require it for survival, or for Ret expression.

### Brn3a is required for the expression of a battery of Runx1-dependent nociceptor markers

Nociceptors can be classified by the expression of peptides such as substance P, calcitonin gene related peptide (CGRP) and somatostatin, and a variety of receptors and channels that function in pain reception and transmission, including G-protein coupled receptors, such as opioid receptors and Mas1-related receptors (Mrgs), cation channels of the transient receptor potential (Trp) family, and the tetrodotoxin-resistant sodium channels Scn10a/Nav1.8 and Scn11a/Nav1.9 [2]. Many of the receptors and channels that mediate nociceptor function in the DRG are dependent on Runx1, while the sensory peptides are not [7]. The mature expression pattern of these sensory markers is not complete until the second postnatal week, but we were able to assess the expression of several of these factors in the TG of P0 Brn3a^-/- ^mice.

**Figure 3:**
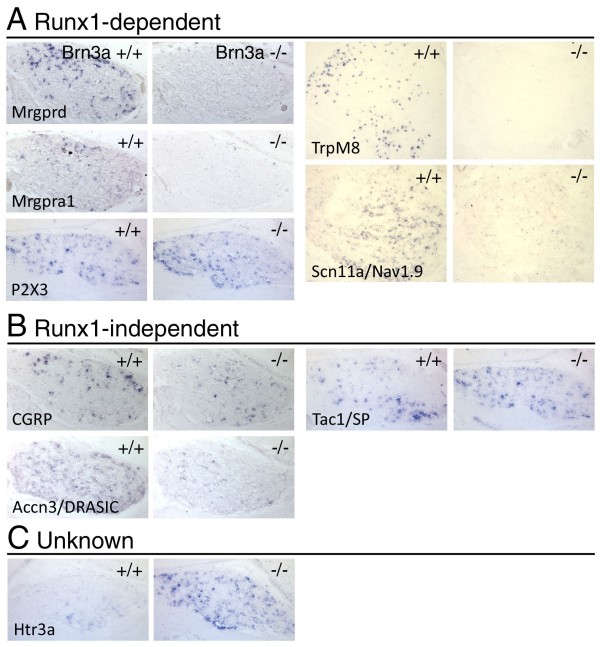
**Expression of Runx1-dependent and Runx1-independent transcripts for receptors, channels and neuropeptides in the Brn3a^-/- ^TG**. The TG of Brn3a^-/- ^and control mice were examined for the expression of multiple sensory markers at P0, the last stage available due to the neonatal death of the knockout mice. Coronal sections are shown except for Trpm8 and Scn11a, which are horizontal sections. **(A) **Expression of transcripts showing Runx1-dependence in the DRG. **(B) **Expression of transcripts independent of Runx1 in the DRG. **(C) **Expression of transcripts for which Runx1-dependence has not been characterized.

The TG of Brn3a^-/- ^newborn mice showed a profound reduction of most Runx1-dependent channels and receptors (Figure 3A). Expression of TrpM8 was undetectable, and Mrgprd, Mrgpra1, and Scn11a were markedly reduced. In contrast, expression levels of mRNA for the Runx1-independent neuropeptides CGRP and Substance P/Tachykinin 1 (SP/Tac1) were little changed (Figure 3B), consistent with past results [16,17]. Although we have previously shown increased expression of somatostatin in the TG of Brn3a^-/- ^embryos at E13.5 [26], in accordance with a previous study [16], we found that somatostatin was not detectable in the wild-type or knockout ganglion at P0 (data not shown).

Changes in the expression of some transcripts did not closely parallel the effects of Runx1 deletion in the DRG. The ATP receptor P2X_3 _is found primarily in non-peptidergic nociceptors in the DRG, but associated with a variety of sensory neuron types in the TG [41]. P2X_3_-expressing neurons are markedly reduced in Runx1 knockout DRG, but little changed in the Brn3a^-/- ^TG (Figure 3A). Expression of the acid-sensing receptor Accn3/DRASIC, also found in a range of large and small sensory neurons in the TG [42], is Runx1-independent in the DRG and was markedly decreased in the Brn3a^-/- ^TG (Figure 3B). These differences may be due to the presence of distinct nociceptor populations in the DRG and TG, or represent Runx1-independent Brn3a targets (see Discussion).

The only mediator of nociception found to show a marked increase in expression in the Brn3a^-/- ^TG was the serotonin receptor subunit Htr3a (Figure 3C), which, unlike other serotonin receptors, is a ligand gated ion channel that forms functional pentameric 5HTR_3 _complexes with itself or Htr3b subunits [43]. It is expressed by a distinct class of nociceptive neurons, most of which do not express SP, and appear to mediate persistent but not acute pain [28]. It may thus characterize a 'nonpeptidergic' class of nociceptors, but these results suggest that it is regulated very differently from canonical non-peptidergic markers.

### Brn3a is required for the initiation of Runx3 expression and maintenance of TrkC

**Figure 4:**
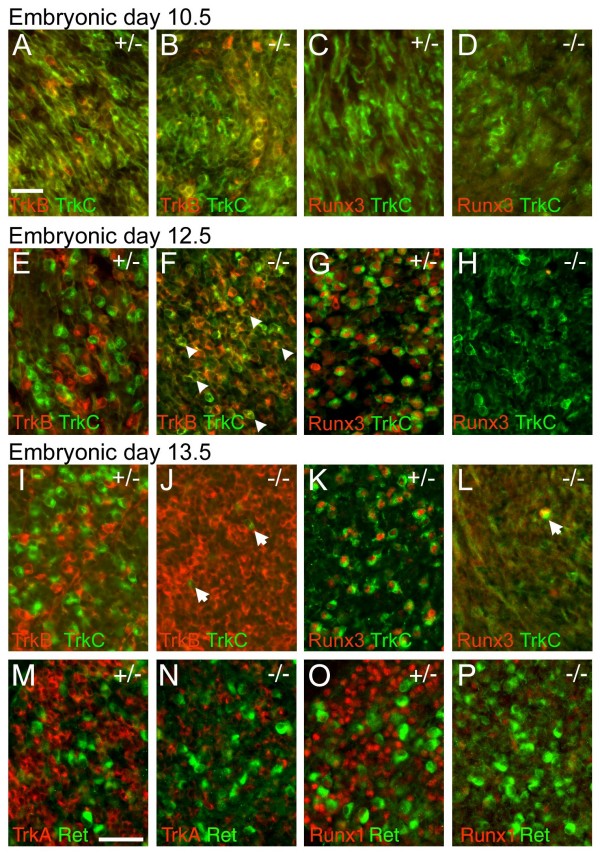
**Early defects in specification of TrkC neurons in the Brn3a^-/- ^TG**. The TG of Brn3a knockout and control embryos were examined at various stages for the expression of TrkB, TrkC and Runx3. **(A-D) **At E10.5 TrkB and TrkC are extensively co-expressed in both genotypes. Runx3 was not detected at this stage in either genotype. **(E-H) **At E12.5 TrkB and TrkC expression identify discrete subsets of neurons in control ganglia (E), but in Brn3a knockout ganglia co-expression persists (F), and examples of the numerous co-expressing neurons are indicated by arrowheads. Runx3 and TrkC are co-expressed in control ganglia (G), but Runx3 expression is not initiated in the knockout TG, and TrkC expression is diminished (H). **(I-L) **At E13.5, TrkB expression is markedly expanded in knockout ganglia (J), and TrkC and Runx3 expression is nearly absent (J, L). Rare remaining TrkC^+^/Runx3^+ ^neurons in the knockout TG are indicated by arrows (J, L). **(M-P) **Early Ret^+ ^neurons at this stage do not co-express TrkA or Runx1 in the control or knockout ganglia, and persist in Brn3a^-/- ^ganglia. Ret^+ ^neurons at this stage are likely to represent a subset of mechanoreceptors; Ret^+ ^nociceptors are not detected until the perinatal period. Scale bar = 50 μM.

We next looked at the timing of the expression of TrkC and Runx3 markers in Brn3a^-/- ^and control embryos to determine at what point in the development of these neurons Brn3a is required. TrkC is widely expressed in the TG at E10.5, and overlaps TrkB at this stage (Figure 4A). It is clear that Brn3a is not required for the initiation of TrkC expression because at E10.5 TrkC is expressed appropriately in Brn3a^-/- ^embryos, and is co-expressed with TrkB in a pattern similar to the wild type (Figure 4B). In contrast, Runx3 is not expressed at this stage in either genotype (Figure 4C, D).

By E12.5, TrkB- and TrkC-expressing neurons normally segregate into a non-overlapping pattern (Figure 4E). Runx3 is expressed in the trigeminal at this stage, and its expression pattern is nearly congruent with that of TrkC (Figure 4G). In Brn3a^-/- ^embryos, TrkB- and TrkC-expressing neurons do not appropriately segregate into distinct populations, and Runx3 expression is not initiated (Figure 4F, H). Discrete populations of TrkB and TrkC/Runx3 expressing neurons are maintained in wild-type ganglia at E13.5 (Figure 4I, K), and throughout development. In Brn3a^-/- ^embryos at E13.5, TrkB is increased relative to controls (Figure 4J) and TrkC is nearly undetectable (Figure 4J, L). In contrast, early-differentiating Ret^+ ^neurons are undiminished in the Brn3a knockout (Figure 4M-P). These early Ret^+ ^neurons do not co-express TrkA or Runx1, either in control or Brn3a^-/- ^TG, and probably constitute a subset of mechanoreceptors [40].

Together these data suggest that permitting the initiation of Runx3 expression is one of the primary roles of Brn3a. The normal initial expression of TrkC in Brn3a^-/- ^TG suggests that regulation of TrkC is a secondary effect. Loss of Runx3 may lead to the failure of TrkB and TrkC to segregate into distinct lineages, consistent with previous work demonstrating that Runx3 represses TrkB expression while promoting expression of TrkC [23,44]. This is then followed by loss of TrkC expression in all TrkB/C precursors by E13.5.

### Brn3a is required for Runx1 expression and the segregation of TrkA/TrkB expression in nociceptor precursors

**Figure 5:**
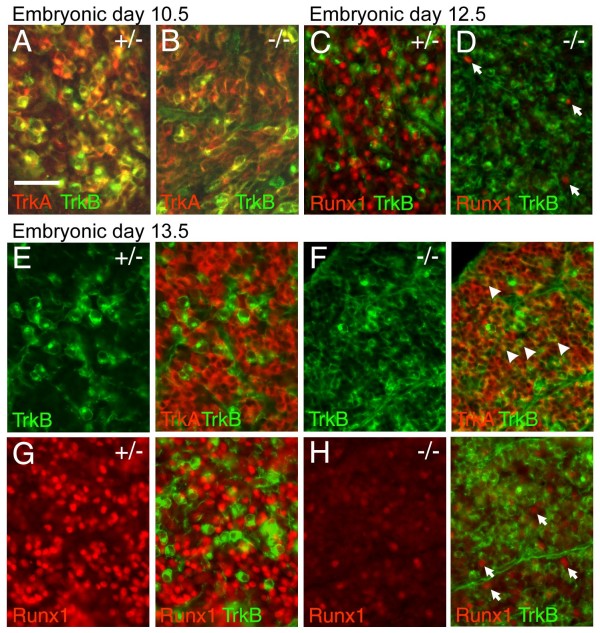
**Defects in nociceptor specification in the Brn3a^-/- ^TG**. The TG of Brn3a knockout and control embryos were examined at various stages for the expression of TrkA, TrkB, and Runx1. **(A, B) **At E10.5, TrkA and TrkB are extensively co-expressed in control (A) and Brn3a knockout (B) ganglia. Runx1 is not expressed at this stage. **(C, D) **At E12.5, Runx1 is expressed in control ganglia (C) but is markedly diminished in the knockout (D). Arrows mark rare Runx1^+ ^cells. **(E, F) **At E13.5, TrkA and TrkB expressing neurons normally segregate into discrete populations (E) but fail to do so in Brn3a knockout ganglia (F). Exposure time for TrkA in (F) is increased to reveal co-expression; see (I, J) for equal exposures. Arrowheads in (F) indicate examples of numerous abnormal TrkA^+^/TrkB^+ ^cells. **(G, H) **At E13.5, Runx1 expression is markedly diminished in the knockout. The few Runx1^+ ^neurons detected at this stage do not express TrkB (H, arrows). Scale bar = 50 μM.

At E10.5, TrkA is co-expressed with TrkB (Figure 5A, B), in a manner similar to TrkC. Co-expression of TrkA and TrkB prior to E11.5 has been observed in prior studies [45]. To identify the stage at which TrkA and TrkB become discrete, we examined embryos at half-day developmental steps from E10.5 to E12.5, and observed that TrkA/B are significantly co-expressed at E11.0, are still co-localized in a small number of TG neurons at E11.5, but are discrete by E12.0 (Additional file 5). At E12.5 in the normal TG, TrkA and TrkB are expressed in distinct neuronal populations (Figure 5E), but in the Brn3a knockout these markers fail to segregate (Figure 5F). This led us to consider whether segregation of TrkA/TrkB expression is regulated by Brn3a via Runx activity, as is TrkB/TrkC expression.

In normal embryos, Runx1 is first detectable at E11.5 (Additional file 6). Runx1 expression is severely attenuated in the TG of Brn3a^-/- ^embryos at E12.5 (Figure 5C, D), and recovers only to a small extent later in development (Figures 5G, H and 2I, J). Thus, the timing of the onset of Runx1 expression and of TrkA/B discrimination, as well as the loss of Runx1 and TrkA/B discrimination in Brn3a knockouts, are consistent with a role for Runx1 in the repression of TrkB in TrkA/B precursors. Further support for a role of Runx1 in restricting the expression of TrkB is derived from the observation that a small number of neurons that maintain Runx1 expression in the Brn3a knockout at E12.5 and E13.5 are TrkB negative (Figure 5D, H). These results demonstrate that Runx1 and Runx3 play similar roles downstream of Brn3a in discriminating sensory subtypes.

### A transactivator Brn3a construct provides insights into its direct targets

Prior microarray studies of the sensory ganglia of Brn3a knockout mice have revealed transcripts both increased and decreased in the absence of this factor [13,26]. However, direct regulation has been demonstrated only for bHLH transcription factors that are expressed early in sensory neurogenesis and subsequently repressed by Brn3a [14], and a direct transcriptional activator function has not been demonstrated for Brn3a *in vivo*. To screen for novel direct targets, we employed the expression of a transgene in which the DNA-binding domain of Brn3a is fused to a strong amino-terminal transactivation domain derived from the herpes virus protein VP16. For genes on which Brn3a functions as a direct repressor, VP16-Brn3a would be expected to phenocopy the knockout because expression of a constitutive transactivator should mimic the loss of a repressor. However, for genes on which Brn3a functions as a direct activator, if any, VP16-Brn3a would be expected to induce changes in gene expression opposite to the loss-of-function mutant.

**Figure 6:**
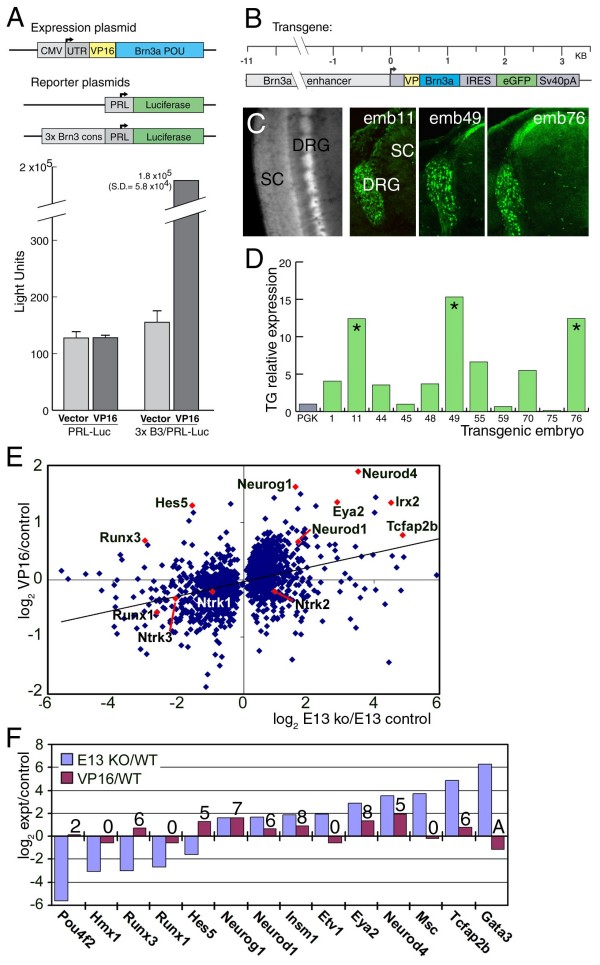
**Sensory-specific expression of a VP16-Brn3a constitutive transcriptional activator reveals possible direct regulatory targets**. **(A) **Activation of transcription by VP16-Brn3a in transfection assays. Increased expression requires both the VP16-Brn3a protein and the presence of a consensus Brn3a binding site (3× Brn3a cons) in the reporter gene construct. **(B) **Structure of the VP16-Brn3a transgene. A dicistronic message encoding VP16-Brn3a and enhanced green fluorescent protein (eGFP) is driven by an 11-kb sensory enhancer from the Brn3a locus. **(C) **Expression of eGFP in the E13.5 dorsal root ganglia (DRG) of VP16-Brn3a transgenic founders by direct GFP fluorescence (whole mount) and immunofluorescence (sections). Appropriate axonal projections to the dorsal spinal cord (SC) are evident. The TG of these embryos were harvested for array analysis. **(D) **RT-PCR assays of Brn3a-VP16 expression in TG dissected from transgenic embryos, normalized to phosphoglycerate kinase (Pgk) expression. Embryos 11, 49, and 76 (asterisks) were selected for microarray analysis. **(E) **Microarray analysis of changes in gene expression in VP16-Brn3a transgenic embryos at E13.5. Changes in gene expression in the VP16-Brn3a transgenic TG were plotted (y-axis) against changes in the E13.5 Brn3a knockout (x axis). Only data for transcripts showing significantly increased or decreased expression in the Brn3a^-/- ^TG relative to controls are shown, thus data points are missing from around the origin. For the entire group, the correlation was weak (slope = 0.126, R^2 ^= 0.147), suggesting that many Brn3a targets are indirectly regulated. The microarray analyses of Brn3a^-/- ^and control ganglia used for comparison purposes will be presented in detail elsewhere (Lanier *et al*., in preparation). **(F) **Changes in transcription factor expression in E13.5 Brn3a knockout ganglia and VP16-Brn3a ganglia, and the number of change calls (out of nine possible comparisons) for VP16-Brn3a versus control ganglia. 'A' indicates absent call. Neurod1 and Neurod4, previously shown to be directly repressed by Brn3a, were activated by VP16-Brn3a. Runx3 and Hes5 are candidates for direct positive regulation by Brn3a (expression increased by VP16-Brn3a, decreased in knockout), Neurog1, Insm1, Eya2, and Tcaf2b are further candidates for direct negative regulation (expression increased by VP16, increased in knockout).

We first tested the function of the VP16-Brn3a fusion protein in heterologous cell transfection assays, where it induced a >1,000-fold activation of a luciferase reporter construct (Figure 6A). Transactivation was entirely dependent on the presence of a Brn3a consensus binding site [29] in the reporter plasmid. A transgene was then constructed containing VP16-Brn3a, an IRES sequence, and a green fluorescent protein (GFP) reporter (Figure 6B). This expression cassette was placed downstream of regulatory sequences derived from the mouse Brn3a locus that have previously been shown to target expression specifically to sensory neurons throughout the neural axis [21]. Transgenic founder embryos were harvested at E13.5, and GFP expression in the DRG was used to verify the specific expression of the transgene in the sensory neurons of individual embryos (Figure 6C). The DRG of the VP16-Brn3a embryos appeared normal in size, and produced the expected axonal projections into the periphery and the superficial lamina of the spinal cord.

Trigeminal ganglia were isolated from those embryos showing strong sensory GFP expression (Figure 6D), and independent microarray analyses (Affymetrix 430v2 mouse microarray) were performed on TG from the three embryos showing the highest expression of the transgene by quantitative PCR. Three littermate embryos, matched for developmental stage, were analyzed in parallel as controls, allowing a total of nine (3 × 3) semi-independent comparisons between VP16-Brn3a and control samples using the Affymetrix MAS5 analysis software. The magnitude of the changes in gene expression in the VP16-Brn3a mouse were modest compared to those seen in the knockout. The effects of VP16 misexpression may be limited because the transgene was not expressed in all TG neurons, and because the VP16-Brn3a protein must compete with endogenous Brn3a for target gene site occupancy. Additional file 1 provides a complete list of gene expression changes in the VP16-Brn3a embryo for all transcripts showing a greater than three-fold change in the Brn3a knockout. Those transcripts with an increased (I) call in greater than half of the nine two-way MAS5 comparisons are highlighted and represent potential direct targets of regulation by Brn3a.

We then plotted the changes in gene expression in the VP16-Brn3a mouse against changes observed in Brn3a knockout TG (Figure 6E), revealing similar regulation of key developmental transcription factors (Figure 6F). Because the direct effects of VP16-Brn3a result only in gene activation, the decreased expression of any gene is presumed to be an indirect effect. Thus, all transcripts below the x-axis in this plot are probably not direct targets of Brn3a at this developmental stage, and notably this group includes Runx1 and all the Trk-class receptors.

Transcripts in the top right quadrant of Figure 6E show higher than wild-type expression in both the Brn3a knockout and in the VP16-Brn3a mouse (that is, the VP16 fusion phenocopies the knockout), indicative of a role for Brn3a in the direct repression of these genes. This group contains many transcription factors, including the known directly repressed targets *Neurod1 *and *Neurod4 *[14]. Other potential targets of direct repression revealed by this screen include *Neurog1 *(neurogenin 1) and the homeodomain factor Irx2.

Those transcripts in the top left quadrant are decreased in the knockout but activated by VP16-Brn3a and are thus good candidates for genes directly activated by Brn3a. Remarkably few genes show this relationship, suggesting that Brn3a functions primarily as a repressor at this stage. Two of the most prominent transcripts in this category are Hes5 and Runx3, and *Runx3 *was selected for further studies of direct regulation by Brn3a.

### Identification of occupied Brn3a binding sites in the *Runx3 *locus

**Figure 7:**
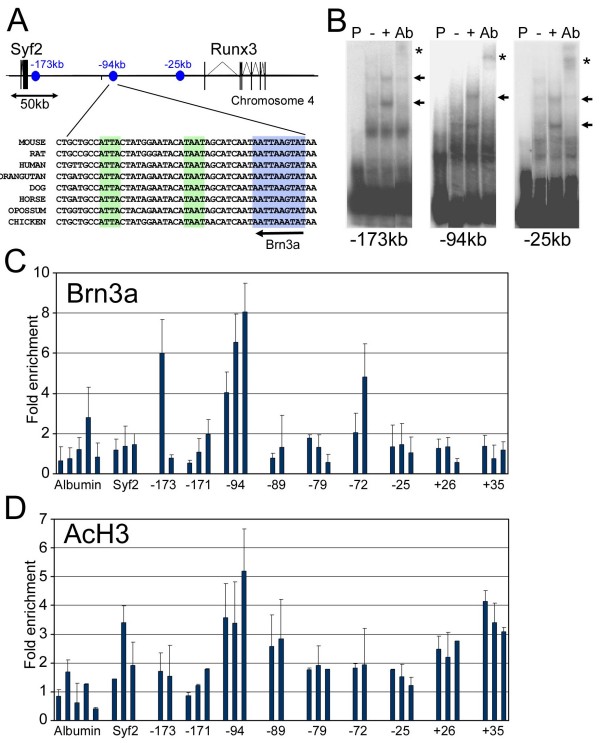
**Brn3a binds to regulatory sequences within the Runx3 locus**. **(A) **Map of a 300-kb region of mouse chromosome 4 including the *Runx3 *and *Syf2 *loci. The location of three sites (-173 kb, -94 kb and -25 kb) showing strong *in vitro *Brn3a binding are indicated (blue circles). Also shown is an interspecies sequence alignment of a region within the proposed -94-kb enhancer, which contains a conserved consensus Brn3a binding site (blue box) and additional conserved TAAT motifs (green boxes) that represent additional potential homeodomain transcription factor binding sites. **(B) **EMSA for Brn3a binding to sites identified by sequence search; assays for the three sites exhibiting strongest binding are shown. P, probe only; -, probe incubated with protein extract from untransfected HEK cells; +, probe incubated with protein extract from HEK cells transfected with a Brn3a expression construct; Ab, Brn3a-transfected cell extract plus supershifting anti-Brn3a antibody; arrow, shifted band; asterisk, supershifted band. Multiple shifted bands may indicate cooperative binding of Brn3a to adjacent sites [56]. **(C) **ChIP profiling of *in vivo *Brn3a binding to the Runx3 locus in E13.5 TG. Two or three primer pairs were made to the regions containing each of the nine potential Brn3a binding sites identified by sequence search (Additional file 4). Quantitative PCR assays were performed on immunoprecipitated chromatin selected with an anti-Brn3a antibody from three independent samples. Fold enrichment for selected/unselected was calculated using the cycle threshold difference method (Methods). Graph shows the mean + standard error for three assays of independent samples. As expected, binding is not observed at the promoter region of the *Alb *(serum albumin) locus, which is not transcribed in neurons and does not contain Brn3a binding sites [14]. Unpaired *t*-test for selection at -94-kb site: n = 8 experimental assays, n = 13 alb locus controls, *P *= 7 × 10^-5^. **(D) **ChIP profiling of histone H3 acetylation at the *Runx3 *locus. Assays were performed as described in (C), with an antibody recognizing lysine 9/14 acetyl histone H3. Increased acetylation in the vicinity of the Brn3a binding site at -94 kb suggests an open conformation of chromatin at this location.

*Runx3 *is a large gene locus consisting of six exons spanning 57 kb, and lying 183 kb from its nearest 5' neighbor, *Syf2 *(Figure 7A). We have previously employed a locus-wide ChIP assay to determine Brn3a binding sites in the *Neurod1 *and *Neurod4 *loci [14]. However, the size of the *Runx3 *locus and its flanking sequences make this approach problematic. Instead we used existing information about Brn3a DNA binding sites to identify potential sites in the *Runx3 *locus that could then be subsequently tested using EMSA and ChIP assays.

Brn3a is a homeodomain-containing transcription factor with a bipartite binding site, which in general binds to sites containing two TAAT motifs in close proximity. Brn3a has been shown to have an optimal binding site of ATAATTAAT but also recognizes sequences with conservative substitutions conforming either to the consensus AT^A^/_T_A^A^/_T_T^A^/_T_AT or having a single base difference from the optimal site at specific positions [29]. Binding sites in the *Neurod1 *and *Neurod4 *loci occupied by Brn3a in the developing TG have exactly matched this consensus [14]. We searched the *Runx3 *locus for potential binding sites within these parameters, and identified ten candidate sites (Additional file 2). Two of these sites are located within introns of the *Runx3 *gene, while the remaining eight are located up to 173 kb upstream of the *Runx3 *transcriptional start site. Brn3a binding to the identified sites was tested *in vitro *using EMSA with oligonucleotide probes and cell lysates from Brn3a-transfected HEK293 cells. The three sites that exhibited the strongest *in vitro *binding were located at -173 kb, -94 kb and -25 kb relative to the *Runx3 *transcription start site (Figure 7B).

Because we have previously shown that *in vitro *binding of Brn3a to a specific regulatory element is a necessary but not sufficient requirement for *in vivo *site occupancy [14], we next used ChIP to assay Brn3a binding to its *Runx3 *locus recognition sites in the TG. E13.5 was chosen for these assays, 1 to 2 days after the initiation of Runx3 expression, because we reasoned that at this time point regulators of Runx3 expression would still be bound to their targets. Two or three PCR primer pairs were designed to the immediate vicinity of the ten potential Brn3a sites (Additional file 4), and enrichment of the target sequences in Brn3a-selected samples relative to an unselected (input) sample was determined using the cycle threshold difference method [32]. Five primer sets designed to the *Alb *locus (serum albumin), which is silent in the sensory ganglia and does not contain Brn3a binding sites [14], were used to normalize the enrichment values from *Runx3*. Additional control primers were designed to sequences within the *Syf2 *gene, which lies 183 kb 5' to the of *Runx3 *transcriptional start site (Figure 7A).

The majority of sites tested gave ChIP enrichment values of two-fold or less relative to the *Alb *locus, indicating that these sites are probably not occupied by Brn3a *in vivo*. The *Syf2 *primers also yielded enrichment values close to baseline. Notably, a site at -25 kb, which bound Brn3a efficiently *in vitro*, did not appear to be occupied *in vivo*. Enrichment was observed for some primers encompassing the binding sites at -173 kb and -72 kb, but consistent selection was observed only for the compound binding site located at -94 kb, which showed a mean enrichment of six-fold. The region encompassing this site shows strong cross-species conservation among several eutherian mammals, opossum, and chicken (Figure 7A). In addition, the region contains multiple TAAT motifs that may accommodate multimeric Brn3a binding or transcriptional partners.

Regulatory regions of chromosomes are known to be associated with histone modifications, including acetylation of H3 subunits, which are believed to induce an open form of chromatin accessible to transcription factors [46,47]. In prior work we have demonstrated that binding of Brn3a autoregulatory regions in the *Pou4f1 *locus and to downstream target gene loci is highly correlated with H3 acetylation [14]. We therefore examined the *Runx3 *locus and control loci by ChIP to assess the histone H3 acetylation state of the potential Brn3a binding sites (Figure 7D). Peak enrichment (5.2-fold) was observed at the identified binding site at -94 kb. In contrast, the region containing the Brn3a consensus site at -25 kb was not significantly H3 acetylated, which may explain why it is not occupied by Brn3a.

## Discussion

### Cell loss in the Brn3a knockout TG

The results presented here for the populations of TG neurons present at birth in Brn3a^-/- ^mice differ somewhat from prior studies. We find approximately 72% of TG neurons are present at birth, compared to more profound cell losses noted by others [10,18,33]. Our results for stage-specific changes in neurotrophin expression are consistent with prior studies showing early loss of TrkC expression [11,18], abnormally increased expression of TrkB at E12.5 to E13.5, and late loss of TrkA [18]. However, prior studies have reported nearly complete elimination of the TrkB^+ ^population by birth [18,33], whereas here we observe a significant population of TrkB^+ ^neurons, exhibiting abnormal morphology, persisting at P0 (Figure 2H). Thus, the most likely explanation for the relative preservation of cell number in the Brn3a^-/- ^TG observed here is the delayed elimination of an abnormal TrkB^+ ^population of neurons, perhaps due to differences in genetic background. We infer that if Brn3a^-/- ^mice survived after birth, the results of these studies would converge in the early postnatal period.

### Brn3a is required for sensory subtype differentiation, via promotion of Runx expression

Early sensory neurons express multiple Trk receptors, and depend on the activity of Runx transcription factors to refine this non-specific pattern into mutually exclusive TrkA, TrkB and TrkC expression. For example, TrkC^+ ^DRG proprioceptors develop from TrkB^+^/TrkC^+ ^precursors, a subset of which initiate expression of Runx3, followed by downregulation of TrkB and exclusive expression of TrkC in the mature neuron [23]. In Runx3^-/- ^embryos, TrkC expression is lost, coupled with an increase in TrkB^+ ^neurons, while mis-expression of Runx3 promotes TrkC and represses TrkB [23,34]. Runx3 repression of TrkB expression appears to be mediated by direct binding of Runx3 to the *Ntrk2 *locus [44].

Runx1 is initially co-expressed with TrkA in the nociceptor lineage [7,8], and initiation of TrkA expression is not dependent on Runx1 [48]. The emphasis of recent work on Runx1 has been on its role in late nociceptor development, and the effect of the loss of Runx1 on the initial discrimination of TrkA/TrkB-expressing precursors has not been reported. However, ectopic expression of Runx1 in E12.5 DRG represses TrkB expression, promotes both TrkA and TrkC, and appears to be interchangeable with Runx3 in these effects [23]. Thus, it is likely that both Runx1 and Runx3 function to repress TrkB and promote the exclusive expression of TrkA and TrkC in nociceptors and proprioceptors, respectively.

The Brn3a^-/- ^TG exhibits a profound decrease in the expression of both Runx3 and Runx1, and failure to appropriately activate Runx expression can account for much of the early developmental phenotype of the Brn3a^-/- ^TG. In control TG, Runx3 expression follows TrkC by one developmental day. In Brn3a^-/- ^TG, TrkC expression is initiated normally, although Runx3 is never expressed, indicating that Runx3 is necessary for the maintenance but not the onset of TrkC expression, consistent with prior results in Runx3^-/- ^embryos [34]. By E13.5, when the expression of TrkA/TrkB/TrkC should be discrete, there is extensive ectopic expression of TrkB in the Brn3a knockout. The abnormally persistent co-expression of TrkB and TrkC in the Brn3a^-/- ^TG, and the subsequent loss of TrkC expression, are all consistent with effects mediated by Runx3.

Brn3a^-/- ^TG neurons also exhibit abnormal persistence of the co-expression of TrkA and TrkB, and subsequent loss of TrkA expression. Given the ability of Runx1 to repress TrkB [23], it is very likely that the failure to discriminate TrkA^+ ^and TrkB^+ ^neural identities in the Brn3a^-/- ^TG is due to the loss of Runx1 function, and that Runx1 performs an analogous function in nociceptor precursors to that of Runx3 in the TrkC lineage. TrkB expression may represent a default pathway adopted by trigeminal neurons in the absence of Runx-mediated repression.

Nakamura *et al*. [49] have observed that the DRG of Runx3 knockouts lack early TrkC^+ ^proprioceptors, but that some TrkC^+ ^neurons appear after E14.5. These late TrkC neurons project cutaneously and co-express TrkA and CGRP. No significant late appearance of TrkC-expressing neurons was observed in the Brn3a^-/- ^TG, and the rare TrkC neurons observed at P0 are Runx3^+^, and have large soma characteristic of proprioceptors or mechanoreceptors (Figure 2B). This suggests that Runx1 promotes TrkC expression in late-developing cutaneous neurons in Runx3 knockouts, but not in Brn3a^-/- ^ganglia in which both Runx1 and Runx3 expression are severely attenuated.

### Brn3a is required for the expression of a battery of functional genes in differentiated nociceptors

Mature nociceptors can be broadly differentiated into peptidergic and non-peptidergic classes, with distinct nociceptive functions and central projections [50]. Peptidergic nociceptors express sensory neuropeptides such as CGRP and SP, while non-peptidergic nociceptors are characterized by the cell-surface marker IB4 [2,3]. The maturation of these nociceptor phenotypes is not complete until the second postnatal week. Recent work has shown that peptidergic nociceptors maintain postnatal expression of TrkA, while non-peptidergic neurons make a late, Runx1-dependent switch from TrkA to Ret expression [7]. The expression of multiple sensory markers, including Trpa1, TrpC3, Trpm8, Scn11a and several members of the Mrg family, are Runx1-dependent in the DRG. Expression of Trpv1 and Trpv2 is affected only in a subset of high-expressing neurons, while SP, CGRP, Accn3 and the mu opioid receptor (MOR) exhibit normal or increased expression in Runx1 knockouts [7,48]. The role of Runx1 in determining the final nociceptor gene expression profile is complex, in that the loss of Runx1 favors neuropeptide expression, and CGRP is repressed by ectopic expression of Runx1 [23], yet some functional genes associated with the peptidergic phenotype, such as Trpm8 and TrpA1, are Runx1-dependent [3].

The TG of Brn3a^-/- ^newborn mice exhibit marked reduction in Trpm8, Mrga1, Mrgd and Scn11a, while CGRP and SP are minimally affected. These results are consistent with the hypothesis that much of the Brn3a^-/- ^nociceptor phenotype is mediated by the loss of Runx1. However, differences were noted between the Brn3a^-/- ^TG and published results for the Runx1 knockout for the purine receptor P2X_3_, which is reduced in Runx1^-/- ^DRG but shows little change in the Brn3a^-/- ^TG, and the acid-sensitive channel Accn3, which is increased in Runx1^-/- ^DRG but decreased in the Brn3a knockout. P2X_3_-expressing sensory neurons are heterogeneous in the TG, and include medium and large neurons with myelinated axons as well as the IB4-positive non-peptidergic nociceptors that predominate in the DRG [41]. Thus, it is likely that differences between the Brn3a and Runx1 knockouts with respect to P2X_3 _expression result from differing effects on distinct populations of P2X_3_^+ ^neurons in the TG and DRG. Accn3 is also expressed in small peptidergic and non-peptidergic neurons and as well as mechanorecptors [42], and may also identify different cell populations in the DRG and TG.

Ichikawa *et al*. [17] have previously reported, consistent with the present study, that CGRP expression is minimally changed in the Brn3a^-/- ^TG. They have also shown that mu opioid receptor (MOR) expression is increased, and that Trpv1 expression is present in small- and medium-sized neurons, and is unchanged [16]. These results are also consistent with changes mediated by the loss of Runx1 in Brn3a knockouts.

Runx1-mediated effects are not sufficient to explain the loss of TrkA expression in the Brn3a^-/- ^TG. Early expression of TrkA is normal in the Brn3a knockouts (Figure 5A, B) but begins to decline at mid-gestation and it is markedly reduced by birth in the TG (Figure 2N) [18] and in the DRG [11]. In contrast, Runx1^-/- ^DRG show increased numbers of TrkA^+ ^neurons [7]. Brn3a has been reported to directly regulate the expression of TrkA [33,39,51], and this may occur independently of the Runx1-mediated changes in gene expression.

### Brn3a directly regulates Runx3 expression

Nearly complete loss of Runx3 in the Brn3a^-/- ^TG from the time of normal onset of its expression is suggestive of direct regulation. Runx3 is one of a small number of genes activated by VP16-Brn3a that are also decreased in the Brn3a knockout, indicating that Brn3a acts primarily as a transcriptional repressor in the early phase of TG development. Brn3a has previously been shown to directly repress the expression of Neurod1 and Neurod4 [14] but an activator function has, to date, only been demonstrated in cell transfection models [21,29].

Here we have shown that Brn3a interacts directly *in vivo *with an enhancer element located approximately 94 kb upstream of the *Runx3 *transcriptional start site. Although this enhancer is distant from the transcription start site, it is evolutionarily conserved and bears histone modifications associated with open chromatin that have been found at other *in vivo *Brn3a binding sites [14]. It is not unusual for regulatory elements in developmental genes to be located at large distances from their respective transcription start sites. Examples include regulatory elements 400 kb from the promoter of sonic hedgehog (*Shh*) [52] and an enhancer element 210 kb downstream of the *Pax6 *promoter [53]. ChIP assays provide less consistent evidence for Brn3a binding to two other regions of the *Runx3 *locus, and other sites may exist that were not predicted by the consensus recognition sequence used and thus were not tested in this study.

### Pan-sensory transcription factors and subtype specification

Because it is a pan-sensory transcription factor, Brn3a is not a good candidate for the initial specification of sensory subtypes. Instead, Brn3a allows trigeminal neurons to progress from a ground state in which all three neurotrophin receptors are co-expressed to a differentiated state in which they are discrete. Brn3a creates a permissive condition for subtype specification by promoting Runx1 and Runx3 expression, which then refine sensory neuron phenotypes by repressing TrkB in prospective TrkA- and TrkC-expressing neurons, respectively. Although Brn3a binds directly to a Runx3 enhancer element, it is clear that this is a necessary but not sufficient condition for the initiation of Runx3 expression since Brn3a is widely expressed at the onset of Runx3 expression, yet Runx3 is only initiated in a subset of Brn3a neurons. It is highly likely that other signals and transcription factors are necessary to confer specificity on Runx3 activation by Brn3a.

The essential role of pan-sensory transcription factors in subtype specification is underscored by the related roles of two other factors in sensory development, the LIM-homeodomain factor Islet1 and the zinc finger protein Klf7, which, like Brn3a, are expressed in all or nearly all sensory neurons prior to E12.5 [9,33]. The DRG of Islet1 conditional knockout mice fail to initiate Runx1 expression. However, unlike Brn3a knockouts, there is a marked decrease in DRG cell number by E13.5. Large Runx3^+^/TrkC^+ ^proprioceptors are maintained in the knockouts, apparently because Runx3^+ ^neurons downregulate Islet1 expression at an early developmental stage and subsequently develop by an Islet1-independent pathway. Klf7, in contrast, does not appear to be necessary for the initial segregation of sensory subtypes, but instead works synergistically with Brn3a to maintain TrkA expression from mid-gestation onward [33]. Brn3a, Islet1, and Klf7 may functionally interact, but are regulated independently, as Brn3a and Islet1 do not regulate one another's expression [54], nor do Brn3a and Klf7 [33]. Together these results fulfill an early prediction that pan-sensory transcription factors would be shown to have pivotal roles in sensory neuron development [55].

## Conclusions

Prior work has shown that Brn3a is necessary to terminate the expression of early neurogenic transcription factors such as Neurod1 and Neurod4 in the developing TG. Here we show that Brn3a also plays a role in sensory subtype specification by promoting the expression of Runx1 and Runx3 in E11.5 to E13.5 ganglia. In Brn3a knockout TG the Runx factors are markedly downregulated, leading to a failure to repress TrkB expression in TrkA- and TrkC-expressing neurons. At birth, the TG of Brn3a knockout mice have multiple defects in the expression of sensory neurotransmitters, channels and receptors, several of which are consistent with known effects of the Runx factors, but some of which are likely mediated by other transcriptional effects of Brn3a. Brn3a activation of the *Runx3 *gene appears to be mediated through direct binding to conserved upstream regulatory elements, constituting the first verified direct transactivator function for this factor.

## Abbreviations

bHLH: basic helix-loop-helix; CGRP: calcitonin gene related peptide; ChIP: chromatin immunoprecipitation; DRG: dorsal root ganglia; E: embryonic day; EMSA: electrophoretic mobility gel shift assay; GFP: green fluorescent protein; P: postnatal day; SP: Substance P; TG: trigeminal ganglia.

## Competing interests

The authors declare that they have no competing interests.

## Authors' contributions

IMD conceived the project and generated most of the data. JL and SRE provided substantial amounts of independent data. IMD and EET wrote the manuscript. EET obtained funding for the project, guided the experiments, and coordinated the inputs of the other authors.

## Supplementary Material

Additional file 1 **Table S1**. **(A) **Potential targets of direct Brn3a repression: VP16 activation of transcripts increased more than three-fold in Brn3a knockouts. **(B) **Potential targets of direct Brn3a activation: VP16 activation of transcripts increased more than three-fold in Brn3a knockouts. Click here for file

Additional file 2 **Table S2**. Probes used for Runx3 locus EMSA. Click here for file

Additional file 3 **Table S3**. Quantitative PCR primers used in the chromatin precipitation assay of the *Runx3 *locus. Click here for file

Additional file 4 **Figure S1: Expression of TrkB mRNA at P0**. Brn3a^+/+ ^and Brn3a^-/- ^newborn mice were harvested at P0 and sectioned in the horizontal plane. *In situ *hybridization for TrkB mRNA shows that TrkB expression is reduced, but present at this stage, consistent with immunofluorescence data for TrkB protein. Click here for file

Additional file 5 **Figure S2: Developmental segregation of TrkA and TrkB expression**. The TG of control embryos were examined at E11, E11.5, and E12.0 in the horizontal plane. TrkA/B immunoreactive cells could no longer be detected at E12. Arrows indicate position of representative TrkA/B co-expressing cells. Click here for file

Additional file 6 **Figure S3: Onset of Runx1 expression in the TG**. The TG of control embryos were examined at E11, E11.5, E12 and E12.5 in the horizontal plane. Runx1 immunoreactivity was first detected at E11.5. At this stage occasional cells were observed which co-expressed Runx1 and TrkB (arrows), but co-expression was transient. Very bright cells indicated by arrowheads are blood/vascular artifacts. Click here for file
