# Supplementary material for: Brn3a regulates neuronal subtype specification in the trigeminal ganglion by promoting Runx expression during sensory differentiation
Source: Neural Dev. 2010 Jan 22;5:3. doi: 10.1186/1749-8104-5-3 (PMC2829025; doi:10.1186/1749-8104-5-3)
Supplement: Additional file 1 — Table S1. (A) Potential targets of direct Brn3a repression: VP16 activation of transcripts increased more than three-fold in Brn3a knockouts. (B) Potential targets of direct Brn3a activation: VP16 activation of transcripts increased more than three-fold in Brn3a knockouts. [file 1749-8104-5-3-S1.PDF]

Table S1A: Potential targets of direct Brn3a repression: VP16 activation of transcripts increased >3 fold in Brn3a KO

			Brn3a knockout			VP16 transgene control				VP16 transgene expression				VP16 comparison	
Probe	Gene Symbol	Gene Title	E13.5 WT mean	E13.5 KO mean	E13.5 KO /WT	C05	C54	C67	Contr mean	VP11	VP09	VP76	VP16 mean	I-Calls	VP16 /contr
1451129_at	Calb2	calbindin 2	18	2125	116.7	13	57	9	26	17	73	11	33	0	1.3
1420992_at	Ankrd1	ankyrin repeat domain 1 (cardiac muscle)	5	560	115.5	26	21	35	27	19	19	26	21	0	0.8
1448886_at	Gata3	GATA binding protein 3	45	3402	75.8	19	47	69	45	6	26	30	21	0	0.5
1424596_s_at	Lmcd1	LIM and cysteine-rich domains 1	6	376	66.0	21	51	35	36	22	39	26	29	0	0.8
1425443_at	Tcfap2d	transcription factor AP-2, delta	17	1009	60.4	12	25	35	24	11	34	32	25	0	1.1
1452142_at	Slc6a1	solute carrier family 6 (neurotransmitter transporter, GABA), member 1	17	500	30.1	30	20	19	23	50	29	11	30	0	1.3
**1435670_at**	**Tcfap2b**	**transcription factor AP-2 beta**	**165**	**4856**	**29.4**	**339**	**301**	**98**	**246**	**641**	**440**	**186**	**422**	**6**	**1.7**
1434201_at	Chrdl1	Chordin-like 1	18	517	28.3	59	23	41	41	59	31	18	36	0	0.9
1453072_at	Gpr160	G protein-coupled receptor 160	18	462	25.9	12	5	5	7	6	9	11	9	0	1.2
1441429_at	Irs4	insulin receptor substrate 4	28	668	23.5	32	17	22	24	44	17	34	32	0	1.3
1426298_at	Irx2	Iroquois related homeobox 2	28	642	23.0	31	25	27	28	82	67	61	70	3	2.5
1457164_at	Trpa1	transient receptor potential cation channel, subfamily A, member 1	13	279	21.8	60	9	53	41	6	17	22	15	0	0.4
1417954_at	Sst	somatostatin	117	2354	20.1	83	118	112	104	56	75	102	78	0	0.7
1442210_at	Prox2	prospero homeobox 2	167	2769	16.6	216	214	237	222	126	206	258	197	0	0.9
1448845_at	Rpp25	ribonuclease P 25 subunit	22	370	16.5	8	16	1	8	31	14	22	22	0	2.7
1446715_at	Abca13	ATP-binding cassette, sub-family A (ABC1), member 13	16	255	16.1	43	23	11	26	90	72	12	58	0	2.3
1449528_at	Figf	c-fos induced growth factor	60	935	15.6	101	52	90	81	117	99	113	110	0	1.4
1418417_at	Msc	musculin	283	3701	13.1	484	348	329	387	361	355	323	346	0	0.9
1457424_at	Eya1	eyes absent 1 homolog	72	879	12.1	173	85	76	111	213	160	70	148	2	1.3
1434939_at	Foxf1a	forkhead box F1a	41	485	11.9	103	57	58	73	38	94	43	58	0	0.8
**1436694_s_at**	**Neurod4**	**neurogenic differentiation 4**	**142**	**1618**	**11.4**	**335**	**186**	**109**	**210**	**1424**	**751**	**172**	**782**	**5**	**3.7**
1451620_at	C1ql3	C1q-like 3	73	789	10.9	138	84	84	102	50	58	62	57	0	0.6
1449566_at	Nkx2-5	NK2 transcription factor related, locus 5	52	559	10.8	23	11	43	26	36	31	6	24	0	0.9
1442542_at	Eya4	eyes absent 4 homolog	39	421	10.7	69	18	26	38	72	41	57	57	0	1.5
1437397_at	Prlr	prolactin receptor	53	507	9.6	72	49	41	54	15	49	37	34	0	0.6
1443749_x_at	Slc1a3	solute carrier family 1 (glial high affinity glutamate transporter), member 3	68	642	9.5	173	76	113	121	79	131	159	123	0	1.0
1447825_x_at	Pcdh8	protocadherin 8	192	1655	8.6	206	228	148	194	320	159	148	209	4	1.1
1437403_at	Samd5	sterile alpha motif domain containing 5	58	455	7.9	135	58	55	83	105	65	74	81	0	1.0

1437268_at	Lancl3	LanC lantibiotic synthetase component C-like 3	34	265	7.8	58	59	23	46	45	25	51	40	0	0.9
**1424127_at**	**Eya2**	**eyes absent 2 homolog**	**158**	**1157**	**7.3**	**203**	**315**	**197**	**238**	**769**	**660**	**400**	**610**	**8**	**2.6**
1439066_at	Angpt1	angiopoietin 1	118	855	7.3	88	102	140	110	183	113	132	143	0	1.3
1440009_at	Olfr78	olfactory receptor 78	122	844	6.9	19	122	64	68	86	72	158	105	2	1.5
1440210_at	Cacng2	calcium channel, voltage-dependent, gamma subunit 2	107	741	6.9	84	138	130	117	209	161	130	167	0	1.4
**1451983_at**	**Irx1**	**Iroquois related homeobox 1**	**130**	**810**	**6.2**	**190**	**153**	**134**	**159**	**343**	**238**	**147**	**243**	**7**	**1.5**
1436465_at	Klhl1	kelch-like 1	72	407	5.6	73	84	79	79	151	80	41	91	0	1.1
1418268_at	Htr3a	5-hydroxytryptamine (serotonin) receptor 3A	1077	5715	5.3	629	959	1,306	965	348	475	933	585	1	0.6
1451260_at	Aldh1b1	aldehyde dehydrogenase 1 family, member B1	136	702	5.2	66	149	124	113	148	123	176	149	0	1.3
1449314_at	Zfpm2	zinc finger protein, multitype 2	280	1362	4.9	257	264	287	269	233	216	244	231	0	0.9
**1452298_a_at**	**Myo5b**	**myosin Vb**	**55**	**267**	**4.8**	**115**	**93**	**63**	**90**	**152**	**169**	**82**	**135**	**5**	**1.5**
**1417928_at**	**Pdlim4**	**PDZ and LIM domain 4**	**360**	**1665**	**4.6**	**662**	**717**	**735**	**705**	**2367**	**1767**	**1031**	**1722**	**9**	**2.4**
1434728_at	Gria3	glutamate receptor, ionotropic, AMPA3 (alpha 3)	81	363	4.5	142	96	73	103	66	110	97	91	0	0.9
1435578_s_at	Dab1	disabled homolog 1	161	659	4.1	169	207	164	180	141	194	170	168	0	0.9
1423630_at	Cygb	cytoglobin	75	305	4.1	116	107	91	105	85	110	127	107	0	1.0
1419874_x_at	Zbtb16	zinc finger and BTB domain containing 16	112	439	3.9	96	83	56	79	77	40	77	65	0	0.8
1450791_at	Nppb	natriuretic peptide precursor type B	132	515	3.9	127	133	144	135	121	140	131	131	0	1.0
1449823_at	Dach2	dachshund 2	166	651	3.9	156	156	142	152	324	170	191	228	2	1.5
1439904_at	Fstl5	follistatin-like 5	661	2555	3.9	446	581	529	519	381	412	483	425	0	0.8
1440487_at	Dcc	deleted in colorectal carcinoma	191	735	3.8	204	140	229	191	241	176	247	221	2	1.2
1425811_a_at	Csrp1	cysteine and glycine-rich protein 1	207	773	3.7	228	294	160	227	394	455	275	375	3	1.6
1422607_at	Etv1	ets variant gene 1	344	1283	3.7	329	336	398	354	184	261	287	244	0	0.7
**1421399_at**	**Insm1**	**insulinoma-associated 1**	**782**	**2863**	**3.7**	**1105**	**1271**	**844**	**1073**	**2584**	**2054**	**1308**	**1982**	**8**	**1.8**
**1455123_at**	**St18**	**suppression of tumorigenicity 18**	**522**	**1906**	**3.7**	**621**	**566**	**460**	**549**	**966**	**984**	**602**	**851**	**7**	**1.5**
1426929_at	Brunol4	bruno-like 4, RNA binding protein	673	2438	3.6	567	738	793	699	277	651	747	558	0	0.8
1453777_a_at	Ndst3	N-deacetylase/N-sulfotransferase (heparan glucosaminyl) 3	80	288	3.6	55	70	53	60	58	52	46	52	0	0.9
**1418086_at**	**Ppp1r14a**	**protein phosphatase 1, regulatory subunit 14A**	**1245**	**4407**	**3.5**	**1560**	**2131**	**1,413**	**1701**	**3538**	**3662**	**1892**	**3031**	**8**	**1.8**
1423835_at	Zfp503	zinc finger protein 503	480	1688	3.5	598	348	343	430	732	404	483	540	2	1.3
1450026_a_at	B3gnt2	UDP-GlcNAc:betaGal beta-1,3-N-acetylglucosaminyltransferase 2	457	1605	3.5	423	459	444	442	432	462	451	448	0	1.0
**1434777_at**	**Mycl1**	**v-myc myelocytomatosis viral oncogene homolog 1**	**198**	**690**	**3.5**	**355**	**291**	**318**	**321**	**603**	**576**	**410**	**530**	**6**	**1.6**
1425846_a_at	Caln1	calneuron 1	95	328	3.5	66	79	76	73	39	33	67	46	0	0.6

1422256_at	Sstr2	somatostatin receptor 2	163	556	3.4	214	145	138	166	141	148	127	139	0	0.8
1423640_at	Synpr	synaptoporin	645	2188	3.4	668	513	488	557	368	313	328	336	0	0.6
1435190_at	Chl1	cell adhesion molecule with homology to L1CAM	465	1567	3.4	251	335	398	328	100	169	239	169	0	0.5
**1430112_at**	**Wdr66**	**WD repeat domain 66**	**90**	**302**	**3.4**	**204**	**145**	**117**	**156**	**622**	**444**	**253**	**440**	**8**	**2.8**
1435435_at	Cttnbp2	cortactin binding protein 2	108	360	3.3	140	99	60	99	135	81	112	109	3	1.1
1417420_at	Ccnd1	cyclin D1	498	1608	3.2	888	595	565	683	1043	852	659	851	4	1.2
**1435941_at**	**Rhbdl3**	**rhomboid, veinlet-like 3**	**115**	**370**	**3.2**	**258**	**239**	**103**	**200**	**331**	**381**	**206**	**306**	**7**	**1.5**
1416612_at	Cyp1b1	cytochrome P450, family 1, subfamily b, polypeptide 1	717	2308	3.2	604	799	845	750	525	624	966	705	1	0.9
**1426413_at**	**Neurod1**	**neurogenic differentiation 1**	**3779**	**12021**	**3.2**	**6281**	**4096**	**3160**	**4512**	**10236**	**7153**	**3963**	**7117**	**6**	**1.6**
1434277_a_at	Ypel2	yippee-like 2	165	524	3.2	230	136	94	153	128	240	158	175	0	1.1
1449519_at	Gadd45a	growth arrest and DNA-damage-inducible 45 alpha	384	1217	3.2	277	342	331	316	370	510	343	408	3	1.3
1418093_a_at	Egf	epidermal growth factor	129	402	3.1	122	128	92	114	190	155	149	165	2	1.4
1435349_at	Nrp2	neuropilin 2	982	3037	3.1	1,104	1,034	1,125	1088	1091	1290	1128	1170	1	1.1
1436791_at	Wnt5a	wingless-related 5A	82	252	3.1	90	63	65	72	61	97	66	74	0	1.0
1420008_s_at	Wwc1	WW, C2 and coiled-coil domain containing 1	276	852	3.1	321	262	333	305	555	665	435	552	4	1.8
1455140_at	Pitpnm3	PITPNM family member 3	151	459	3.0	158	144	178	160	81	126	106	104	0	0.7
1434083_a_at	Elmod1	ELMO domain containing 1	88	268	3.0	148	96	89	111	170	75	68	104	3	0.9
**1438551_at**	**Neurog1**	**neurogenin 1**	**238**	**719**	**3.0**	**607**	**349**	**191**	**382**	**1962**	**1210**	**373**	**1181**	**7**	**3.1**
1429459_at	Sema3d	sema domain (semaphorin) 3D	488	1471	3.0	461	452	411	441	165	245	281	230	0	0.5
1440086_at	Rnf182	ring finger protein 182	204	610	3.0	167	242	278	229	257	159	169	195	0	0.9

Table S1B: Potential targets of direct Brn3a activation: VP16 activation of transcripts increased >3 fold in Brn3a KO

			Brn3a knockout			VP16 transgene control				VP16 transgene expression				VP16 comparison	
**Probe**	**Gene Symbol**	**Gene Title**	**E13.5 WT mean**	**E13.5 KO mean**	**E13.5 KO /WT fold**	**C05**	**C54**	**C67**	**Contr mean**	**VP11**	**VP09**	**VP76**	**VP16 mean**	**I-Calls**	**VP16 /contr fold**
1437588_at	Pou4f2	POU domain, class 4, transcription factor 2; Brn3b	900	19	48.0	837	918	1068	941	1154	886	1113	1051	2	1.1
1424923_at	Serpina3g	serine peptidase inhibitor A3G	1136	27	41.7	770	1047	1461	1092	875	832	1594	1100	2	1.0
1441096_at	Tusc5	tumor suppressor candidate 5	474	17	28.6	436	483	635	518	478	482	707	556	2	1.1
1429667_at	Pou4f1	POU domain, class 4, transcription factor 1; Brn3a (targeted gene)	5818	272	21.4	5524	4501	4499	4841	5629	5508	4568	5235	2	1.1

1449312_at	Npy5r	neuropeptide Y receptor Y5	443	22	20.5	483	305	211	333	346	253	281	293	0	0.9
1421652_at	Htr3b	5-hydroxytryptamine receptor 3B	314	20	15.6	78	158	229	155	48	41	175	88	1	0.6
1430700_a_at	Pla2g7	phospholipase A2, group VII	2497	161	15.5	1376	2361	2797	2178	1225	2047	2870	2047	3	0.9
1438782_at	Cntn4	contactin 4	846	56	15.2	898	698	655	750	472	550	601	541	0	0.7
1417333_at	Rasa4	RAS p21 protein activator 4	307	20	15.0	204	394	419	339	111	289	420	273	1	0.8
1429443_at	Cpne4	copine IV	2647	180	14.7	1399	1945	2367	1904	1401	1855	2442	1900	3	1.0
1424890_at	Bnc1	basonuclin 1	2723	186	14.7	2439	2036	2625	2367	640	1183	1860	1227	0	0.5
1439109_at	Ccdc68	coiled-coil domain containing 68	293	21	13.7	259	199	297	252	350	307	363	340	3	1.4
1448290_at	Pap	pancreatitis-associated protein	615	46	13.3	79	221	480	260	34	3	58	31	0	0.1
1416882_at	Rgs10	regulator of G-protein signalling 10	5547	420	13.2	3875	5446	5384	4902	5593	6301	6910	6268	5	1.3
1421471_at	Npy1r	neuropeptide Y receptor Y1	994	78	12.7	621	909	967	832	240	549	777	522	0	0.6
1419148_at	Avil	advillin	6015	580	10.4	6059	6140	7180	6459	4377	4509	6806	5231	0	0.8
1456543_at	Prokr1	prokineticin receptor 1	678	66	10.2	652	774	682	702	486	444	436	455	0	0.6
1456858_at	Gpr149	G protein-coupled receptor 149	434	45	9.6	157	222	278	219	38	111	215	121	0	0.6
1436876_at	Rgs7bp	regulator of G-protein signalling 7 binding protein	5185	550	9.4	3415	3823	5423	4220	3607	3322	5855	4261	2	1.0
1417956_at	Cidea	cell death-inducing DNA fragmentation factor, alpha subunit-like effector A	411	46	8.9	151	190	203	181	95	129	11	78	0	0.4
1436044_at	Scn7a	sodium channel, voltage-gated, type VII, alpha	3069	359	8.6	2246	2773	2878	2632	2020	1908	2930	2286	0	0.9
1422152_at	Hmx1	H6 homeo box 1	1713	207	8.3	2323	1985	1598	1969	1170	1141	1724	1345	0	0.7
1423856_at	Popdc3	popeye domain containing 3	308	38	8.2	215	284	372	290	297	237	476	336	3	1.2
**1440275_at**	**Runx3**	**runt related transcription factor 3**	**274**	**34**	**8.1**	**421**	**272**	**258**	**317**	**548**	**577**	**403**	**510**	**6**	**1.6**
1455436_at	Diras2	DIRAS family, GTP-binding RAS-like 2	2547	315	8.1	1940	2083	2597	2207	860	1141	1794	1265	0	0.6
1441975_at	Acpp	acid phosphatase, prostate	315	40	7.9	246	325	344	305	55	103	213	124	0	0.4
**1438624_x_at**	**Hs3st2**	**heparan sulfate 3-O-sulfotransferase 2**	**1031**	**135**	**7.6**	**1155**	**808**	**916**	**960**	**2183**	**1118**	**1104**	**1468**	**6**	**1.5**
1460668_at	Gal	galanin	8979	1200	7.5	7743	9297	9270	8770	4408	6466	8566	6480	0	0.7
1437930_at	Glt25d2	glycosyltransferase 25 domain containing 2	343	48	7.1	268	337	356	320	180	320	361	287	0	0.9
1439795_at	Gpr64	G protein-coupled receptor 64	1956	276	7.1	1550	1621	1669	1613	1103	1081	1679	1288	0	0.8
1416956_at	Kcnab2	potassium voltage-gated channel, shaker-related subfamily, beta member 2	2689	398	6.8	2810	3579	3661	3350	2896	3078	3912	3295	0	1.0
1439610_at	Rab27b	RAB27b, member RAS oncogene family	497	77	6.5	387	371	405	388	183	196	381	253	0	0.7
1455324_at	Plcxd2	phosphatidylinositol-specific phospholipase C, X2	4484	695	6.4	4077	3618	4324	4006	2735	2877	3704	3106	0	0.8
1440990_at	Kif26b	kinesin family member 26B	3164	493	6.4	2725	2249	2427	2467	2069	1884	2610	2188	0	0.9
1443392_at	Trpv1	transient receptor potential cation channel, subfamily V, member 1	758	120	6.3	379	672	757	603	251	265	699	405	1	0.7

1422864_at	Runx1	runt related transcription factor 1	1797	286	6.3	975	1254	1280	1170	550	831	994	792	0	0.7
1421836_at	Mtap7	microtubule-associated protein 7	1308	218	6.0	1014	933	1157	1035	528	763	1154	815	0	0.8
1420573_at	Hoxd1	homeo box D1	2987	503	5.9	2614	2215	2543	2457	1910	1655	2092	1886	0	0.8
1430136_at	Grm3	glutamate receptor, metabotropic 3	505	89	5.7	608	382	542	511	739	514	570	607	4	1.2
1425090_s_at	Kcnc4	potassium voltage gated channel, Shaw-related subfamily, member 4	323	57	5.6	274	219	304	266	143	149	159	151	0	0.6
1455450_at	Ptpn3	Protein tyrosine phosphatase, non-receptor type 3	7432	1326	5.6	6309	6737	7137	6728	4966	6198	7292	6152	0	0.9
1425518_at	Rapgef4	Rap guanine nucleotide exchange factor (GEF) 4	903	163	5.5	473	479	518	490	245	379	428	351	0	0.7
1427252_at	Dmrtb1	DMRT-like family B with proline-rich C-terminal, 1	265	49	5.4	231	286	267	261	154	289	270	238	0	0.9
1457632_s_at	Mrg1	myeloid ecotropic viral integration site-related gene 1	6326	1179	5.4	5389	3480	4012	4293	4011	2957	3269	3412	0	0.8
1455226_at	Spnb1	spectrin beta 1	358	67	5.3	225	284	352	287	112	189	307	203	0	0.7
1426501_a_at	T2bp	Traf2 binding protein	557	105	5.3	266	551	1078	632	278	458	907	548	3	0.9
1434800_at	Sv2b	synaptic vesicle glycoprotein 2 b	801	152	5.3	649	826	858	777	571	587	664	607	0	0.8
1455785_at	Kcna1	potassium voltage-gated channel, shaker-related subfamily, member 1	3246	640	5.1	2535	2791	3278	2868	1622	2256	2829	2236	0	0.8
1455328_at	Accn2	amiloride-sensitive cation channel 2, neuronal	2609	519	5.0	2851	2246	4057	3051	2177	2688	2973	2612	0	0.9
1421594_a_at	Sytl2	synaptotagmin-like 2	1139	240	4.7	1009	811	1100	973	435	533	814	594	0	0.6
1436520_at	Ahnak2	AHNAK nucleoprotein 2	450	95	4.7	284	246	367	299	123	175	235	178	0	0.6
1421180_at	Lix1	limb expression 1 homolog	1613	344	4.7	1515	1480	1482	1492	1618	1150	1367	1378	0	0.9
1428443_a_at	Rap1gap	Rap1 GTPase-activating protein	1820	395	4.6	2077	2085	2242	2135	1881	1845	2370	2032	0	1.0
1460214_at	Pcp4	Purkinje cell protein 4	577	126	4.6	217	368	581	388	189	202	453	281	0	0.7
1457589_at	Fat3	FAT tumor suppressor homolog 3	643	142	4.5	520	507	611	546	569	441	581	530	0	1.0
1436532_at	Dclk3	doublecortin-like kinase 3	753	167	4.5	983	937	813	911	840	884	978	901	0	1.0
1452106_at	Npnt	nephronectin	547	121	4.5	243	409	408	353	105	179	287	190	0	0.5
1451985_at	Lrrk1	leucine-rich repeat kinase 1	538	120	4.5	321	492	455	423	489	386	605	493	0	1.2
1434295_at	Rasgrp1	RAS guanyl releasing protein 1	269	61	4.4	172	223	277	224	61	130	166	119	0	0.5
1422353_at	Pou4f3	POU domain, class 4, transcription factor 3; Brn3c	258	60	4.3	384	397	195	325	387	365	422	391	3	1.2
1430291_at	Dock5	dedicator of cytokinesis 5	910	212	4.3	399	685	691	592	431	781	435	549	0	0.9
1434635_at	Rph3a	rabphilin 3A	669	157	4.3	634	640	1101	792	268	292	695	419	0	0.5
1455917_at	Ntrk3	neurotrophic tyrosine kinase, receptor, type 3	1097	261	4.2	1060	1070	1124	1085	482	1072	1056	870	0	0.8
1449536_at	Kcnn1	potassium intermediate/small conductance calcium-activated channel, subfamily N, member 1	317	75	4.2	461	473	624	520	244	357	376	326	0	0.6
1451031_at	Sfrp4	secreted frizzled-related protein 4	410	98	4.2	174	466	345	328	406	288	551	415	3	1.3
1421396_at	Pcsk1	proprotein convertase subtilisin/kexin	266	64	4.2	247	268	297	271	179	184	206	190	0	0.7

		type 1													
1436095_at	Chd5	chromodomain helicase DNA binding protein 5	1037	250	4.1	928	896	1185	1003	393	527	875	598	0	0.6
1448591_at	Ctss	cathepsin S	781	191	4.1	528	628	407	521	773	565	454	597	1	1.1
1457133_at	Tcerg1l	Transcription elongation regulator 1-like	1034	259	4.0	1179	935	1188	1101	920	786	1012	906	0	0.8
1450754_at	Cacna2d2	calcium channel, voltage-dependent, alpha 2/delta subunit 2	2655	676	3.9	2127	2189	2492	2269	3030	2423	2435	2629	1	1.2
1423427_at	Adcyap1	adenylate cyclase activating polypeptide 1	898	231	3.9	619	776	909	768	686	798	910	798	0	1.0
1425503_at	Gcnt2	glucosaminyl (N-acetyl) transferase 2, I-branching enzyme	989	257	3.9	519	613	746	626	278	346	531	385	0	0.6
1436100_at	Sh2d5	SH2 domain containing 5	302	79	3.8	202	254	311	255	118	238	322	226	0	0.9
1423376_a_at	Dok4	docking protein 4	1471	386	3.8	933	1542	1405	1293	552	955	1398	968	1	0.7
1428484_at	Osbpl3	oxysterol binding protein-like 3	746	197	3.8	465	584	943	664	271	361	628	420	0	0.6
1433977_at	Hs3st3b1	heparan sulfate (glucosamine) 3-O-sulfotransferase 3B1	291	77	3.8	199	185	287	224	175	323	175	224	0	1.0
1437401_at	Igf1	insulin-like growth factor 1	3922	1043	3.8	3712	3267	3943	3641	1543	2571	3737	2617	0	0.7
1436954_at	Wipf1	WAS/WASL interacting protein family, member 1	1239	330	3.8	1174	900	883	986	870	1098	938	969	0	1.0
1457032_at	Ak5	adenylate kinase 5	618	165	3.7	428	618	978	675	142	265	508	305	0	0.5
1416069_at	Pfkp	phosphofructokinase, platelet	3105	835	3.7	2134	2876	3461	2824	1174	1999	2856	2010	0	0.7
1418762_at	Cd55	CD55 antigen	382	103	3.7	251	244	319	271	151	219	169	180	0	0.7
1439573_at	Rtn4rl2	reticulon 4 receptor-like 2	855	231	3.7	1160	1102	1074	1112	1506	1347	1361	1405	3	1.3
1429399_at	Rnf125	ring finger protein 125	739	199	3.7	707	593	586	629	425	478	565	489	0	0.8
1419421_at	Ank1	ankyrin 1, erythroid	1169	316	3.7	795	942	1324	1021	684	883	1095	887	0	0.9
1426529_a_at	Tagln2	transgelin 2	1270	352	3.6	2172	1823	1628	1874	2443	1819	1804	2022	2	1.1
1442143_at	Tmem16d	transmembrane protein 16D (eight membrane-spanning domains)	1349	377	3.6	998	943	981	974	560	642	791	664	0	0.7
1449429_at	Fkbp1b	FK506 binding protein 1b	9118	2550	3.6	8419	8264	9560	8748	5639	6069	8171	6627	0	0.8
**1434877_at**	**Nptx1**	**neuronal pentraxin 1**	**221**	**62**	**3.5**	**234**	**144**	**194**	**190**	**432**	**217**	**245**	**298**	**5**	**1.6**
1438452_at	Nebl	nebulette	501	143	3.5	313	508	457	426	311	499	531	447	2	1.0
1450992_a_at	Meis1	myeloid ecotropic viral integration site 1	431	124	3.5	444	351	322	372	257	278	348	294	0	0.8
1438861_at	Bnc2	basonuclin 2	5910	1725	3.4	5730	4099	4732	4854	3030	3184	3861	3358	0	0.7
1459723_at	Zdhhc22	zinc finger, DHHC-type containing 22	2574	752	3.4	2619	2469	2763	2617	1171	1887	2237	1765	0	0.7
1433909_at	Syt17	synaptotagmin XVII	410	120	3.4	176	501	204	293	157	159	447	255	2	0.9
1434096_at	Slc4a4	solute carrier family 4 (anion exchanger), member 4	998	295	3.4	738	1074	1165	992	830	809	1122	920	0	0.9
1455923_at	Kctd8	potassium channel tetramerisation domain containing 8	792	237	3.3	705	547	777	676	838	478	762	693	0	1.0
1443119_at	Grm7	glutamate receptor, metabotropic 7	379	113	3.3	160	252	438	283	83	119	240	147	0	0.5
1428696_at	Rftn1	raftlin lipid raft linker 1	486	147	3.3	287	408	504	399	182	232	385	267	0	0.7

1449921_s_at	Cpne6	copine VI	1384	422	3.3	1025	1455	1669	1383	405	493	941	613	0	0.4
1457780_at	Stx11	syntaxin 11	336	103	3.3	427	308	382	373	227	248	285	253	0	0.7
1417673_at	Grb14	growth factor receptor bound protein 14	2970	913	3.3	2923	2721	2574	2739	2418	2545	2758	2574	0	0.9
1440323_at	Syt2	synaptotagmin II	805	248	3.3	949	910	949	936	582	794	789	721	0	0.8
**1422776_at**	**Serpinb8**	**serine peptdiase inhibitor B8**	**107**	**33**	**3.2**	**89**	**110**	**134**	**111**	**263**	**261**	**159**	**228**	**7**	**2.0**
1435994_at	Kcnh1	potassium voltage-gated channel, subfamily H (eag-related), member 1	1224	386	3.2	1231	1421	1473	1375	865	1044	1377	1095	0	0.8
1444451_at	Pappa2	pappalysin 2	1876	593	3.2	2050	2033	2259	2114	2364	2840	2566	2590	2	1.2
1451931_x_at	H2-L	histocompatibility 2, D region	530	169	3.1	125	496	356	326	233	235	479	316	0	1.0
1427004_at	Fbxo2	F-box protein 2	412	132	3.1	278	332	365	325	99	200	287	195	0	0.6
1417960_at	Cpeb1	cytoplasmic polyadenylation element binding protein 1	606	196	3.1	421	491	450	454	266	399	428	364	0	0.8
1441223_at	4-Mar	membrane-associated ring finger (C3HC4) 4	608	198	3.1	580	596	730	635	104	353	587	348	0	0.5
1428573_at	Chn2	chimerin (chimaerin) 2	1340	440	3.0	1264	1238	1100	1201	1280	1493	1430	1401	3	1.2
1436528_at	Kazald1	Kazal-type serine peptidase inhibitor domain 1	273	90	3.0	214	211	152	193	160	196	250	202	0	1.0
1439725_at	Ptprt	protein tyrosine phosphatase, receptor type, T	483	159	3.0	371	495	458	441	178	208	457	281	0	0.6
1448300_at	Mgst3	microsomal glutathione S-transferase 3	6500	2166	3.0	4235	5137	5498	4957	2774	3458	4013	3415	0	0.7
1451461_a_at	Aldoc	aldolase 3, C isoform	815	272	3.0	530	817	1060	802	358	328	819	501	0	0.6
1448470_at	Fbp1	fructose bisphosphatase 1	439	147	3.0	296	505	664	488	272	293	399	321	0	0.7
1455899_x_at	Socs3	suppressor of cytokine signaling 3	1586	531	3.0	1910	1320	1479	1570	1693	1382	1709	1595	0	1.0
**1455426_at**	**Epha3**	**Eph receptor A3**	**1028**	**345**	**3.0**	**1038**	**921**	**839**	**932**	**1768**	**1273**	**1238**	**1426**	**6**	**1.5**
1419647_a_at	Ier3	immediate early response 3	622	209	3.0	418	555	587	520	294	275	491	353	0	0.7
**1456010_x_at**	**Hes5**	**hairy and enhancer of split 5**	**119**	**40**	**3.0**	**194**	**55**	**63**	**104**	**380**	**294**	**93**	**256**	**5**	**2.5**
1421017_at	Nrg3	neuregulin 3	381	130	2.9	365	263	324	317	310	275	277	287	0	0.9

All genes which showed at least a 3-fold increase or decrease in E13.5 KO trigeminal ganglion, and met specific inclusion criteria (Methods) were examined for changed gene expression in the TG of embryos mis-expressing VP16. Inclusion criteria for transcripts affected by are an I (increase) call in at least 5/9 semi-independent VP16 transgenic vs VP16 control embryos, and a 1.5 fold change in mean expression in this comparision.
