# Supplementary material for: Brn3a regulates neuronal subtype specification in the trigeminal ganglion by promoting Runx expression during sensory differentiation
Source: Neural Dev. 2010 Jan 22;5:3. doi: 10.1186/1749-8104-5-3 (PMC2829025; doi:10.1186/1749-8104-5-3)
Supplement: Additional file 2 — Table S2. Probes used for Runx3 locus EMSA. [file 1749-8104-5-3-S2.PDF]

Table S2. Probes used for Runx3 locus EMSA.

Name	Location	Probe Length	Sequence 5' to 3'<br>Potential consensus and variant binding site(s) underlined
-173	-172,926	40	AGTATGATATTAATTAATTAATTTATATATTTATATGTGG
-171	-170,997	66	TTATTTAAGTTCTGATATTATTATCCTTCCTAGTAGGACCCTAATTAATTAATCATATAGAATCGG
-95	-94,814	54	ATGAATATTTTACAAGAAACCGGAATTATTAATGTTTCAATAAAAACCCAAACC
-94	-94,381	72	TATGGAATACATAATAGCATCAATAATTAAGTATAAATCGGGTTTAATATTTTATGCTGGCGACATGTAATT
-89	-88,761	40	ATAGTGAGACTCAAATAATTAATAATAATAATAGTGTTGT
-79	-79,212	39	ACATGCAGCAAACTGGTGTCATTATAATTAATGCTAGTC
-72	-71,952	40	TAAGGATAGGGTATAGTTAATTATTATTAATACGCGTTAA
-25	-25,378	49	TTAATTAACTAATTAATTAACCAATTAATTAATTAATTAAGGGATGGTT
+26	+26,474	79	CGGCCTCTGAGATTAATTAAAGTTTCACAAGGACTGACAGGCATTAAGACCTTTTTGGAGGGGTGGAAAATCCCTAATA
+35	+35,739	49	TTCAGCATTAATTAAAACCAGGGCGTTATTTGCATGTAATAACTGTTTA

Location of binding sites are given relative to Runx3 transcription start site.
