# Supplementary material for: Brn3a regulates neuronal subtype specification in the trigeminal ganglion by promoting Runx expression during sensory differentiation
Source: Neural Dev. 2010 Jan 22;5:3. doi: 10.1186/1749-8104-5-3 (PMC2829025; doi:10.1186/1749-8104-5-3)
Supplement: Additional file 3 — Table S3. Quantitative PCR primers used in the chromatin precipitation assay of the Runx3 locus. [file 1749-8104-5-3-S3.PDF]

Table S3. qPCR Primers used in the chromatin precipitation assay of Runx3 locus.

Brn3a site	Primer Name	5'Primer	3'Primer
-173	35.3	CGAGGATTCTAGTCTGTCCATG	CAAGTGCTAGAATTGTAGGCAT
	35.5	TGTTTTCTGTGAAAGGACTTGAGG	TCTCTGCACTTTGAATCTGTAGCC
-171	36.5	CATCTGATACTTTAAACTGCGTCCTA	GAACCCCAGTCCTGATGTGT
	37.5	AGCCCTGTCTACCCGCAG	CCAAGCTAGCACAATTAGACCT
	37.9	TGGGCTCTTGAGTCTACCCTGA	TGAGAGACTTTTCCCAGCCAAA
-95, -94	113.7	GGAAATTAAAAGCAGTGGCTGAG	AATATTCATGGTTCCTGCAGCTG
	114	GCTTGGCTTCTTACCTCAAGGA	TCCGTGCCCAGTAAATAGCTTT
	114.2	TGCTGGCGACATGTAATTTACAC	AGACGTGACCTTTGATATCTCGG
-89	119.7	GAAAAGGGCTTCCTACATCAGC	TCATACCTGCCTGGGAAGAAA
	120	ACTAATCCACCTCGGAGAGGG	CCGTCCTTTTAGCTTCTTTCAGC
-79	129	CCTTTAATCCAGAGGAACGCAA	AAACGTCTGCCTTTCTGGTGTC
	129.3	AAGACCAACAGGCACTTTCTCAG	GATCTCCGAAGGCCTGTCACT
	129.6	TATTGCCTAGGGAGGTGTAGCG	CATGAGCCAAAGCGGTTTAACTA
-72	136.5	AGGGAGTTCTGAAACCCAGAGC	ACGGTCAGATGTAGGCCCTAGA
	136.7	CATGGAGTGTTGGAGTAAGCCA	GCTACCTCACAAATTACCCTGCC
-25	183	TAGCACCTGCCTTGTTCATGG	GACCGTGACGTTTTCCTCAAA
	183.3	TAGAGATCCAACCCACCTCTGC	TGCATGCTTTTAATCCCAGCT
	183.5	GAAAGAAAAGTCCAACTCCAGCA	GGGTACGTGGGTGGAAGTAAGA
+26	46.3	CAACTGTTCTCCACCTCGGAAC	TGTACGCATGGCGTGAGATAA
	46.5	AAGTCGTCGGCCTCTGAGATTA	ATGCCTGTCAGTCCTTGTGAAA
	46.8	TCAACAGACCCTTCCCTCCTTA	CGACATTGTCAAATAGCGGATC
+35	56	CCACGCACAGATTTCTTTCAAAC	CATCAACCTGCAACCAGTTAAGG
	55.6	GAGCAATCACCTTCCCAAACTCT	GGCTCACTTCCTAACCCTGGA
	56.1	TGTCATCCCCAATCTCTGAGC	CAGGGCCAGACCTTCAATTAAG
Syf2 locus controls	syf2-2	ATCCTCCTGCCTCTGCCTACTA	GGACTGGGCACTTTAATCCCA
	syf2-3	CTGAAACTCAGGGAGAAGTGGAA	CCTGTGAAGACGGAAGTCAGTTC
	syf2-4	ATAAATACTGGGTGGGTGTGGTG	CCTCCACAGTGCTGGGATAAAA
Alb promoter controls	1 Albumin	GGGATGAACAACCTATGCAATTC	TGGGCCTTGGCATGGA
	2 Albumin	AGAGGTTCATGTTAGATGAAAACATTC TAC	TGTCCCCACCCCATGCT
	Alb1-42	TCCTCCTCTTCGTCTCCGG	CTTACGTGCTTCTCGGCGA
	Alb1-51	TCCATCTGAAGCTTGGGTGTG	GGCCACCTGATGTGCTTTCT
	Alb1-57b	AAATCAACACCCTAAGGAACACAAA	GCAGCACAGAGACAAGAAGTCAA

Note: Binding at sites -94kb and -95kb are too close to be distinguished in ChIP assays.
