# Supplementary figures and images for: Brn3a regulates neuronal subtype specification in the trigeminal ganglion by promoting Runx expression during sensory differentiation
Source: Neural Dev. 2010 Jan 22;5:3. doi: 10.1186/1749-8104-5-3 (PMC2829025; doi:10.1186/1749-8104-5-3)

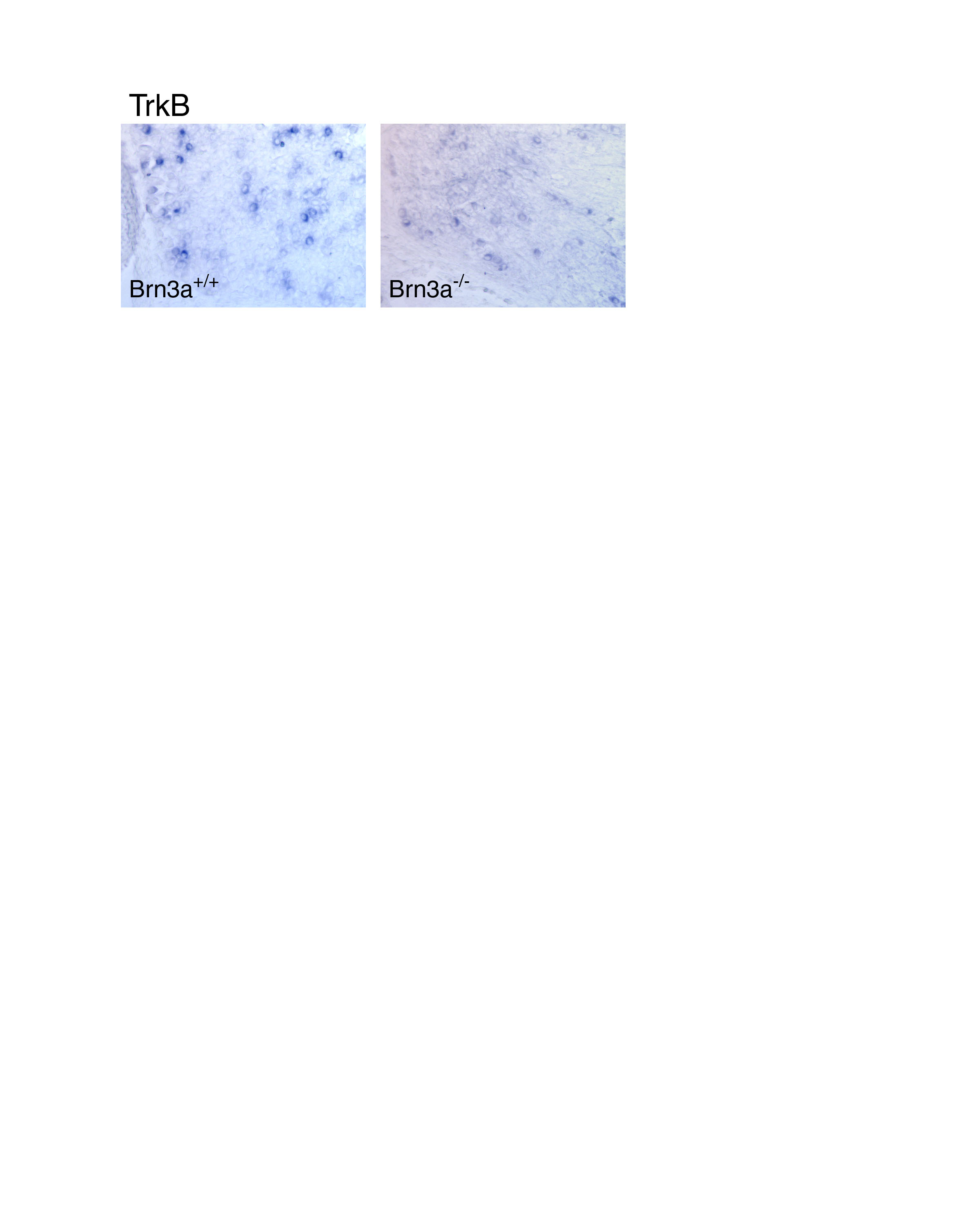

Supplement: Additional file 4 — Figure S1: Expression of TrkB mRNA at P0. Brn3a+/+ and Brn3a-/- newborn mice were harvested at P0 and sectioned in the horizontal plane. In situ hybridization for TrkB mRNA shows that TrkB expression is reduced, but present at this stage, consistent with immunofluorescence data for TrkB protein. [file 1749-8104-5-3-S4.JPEG]

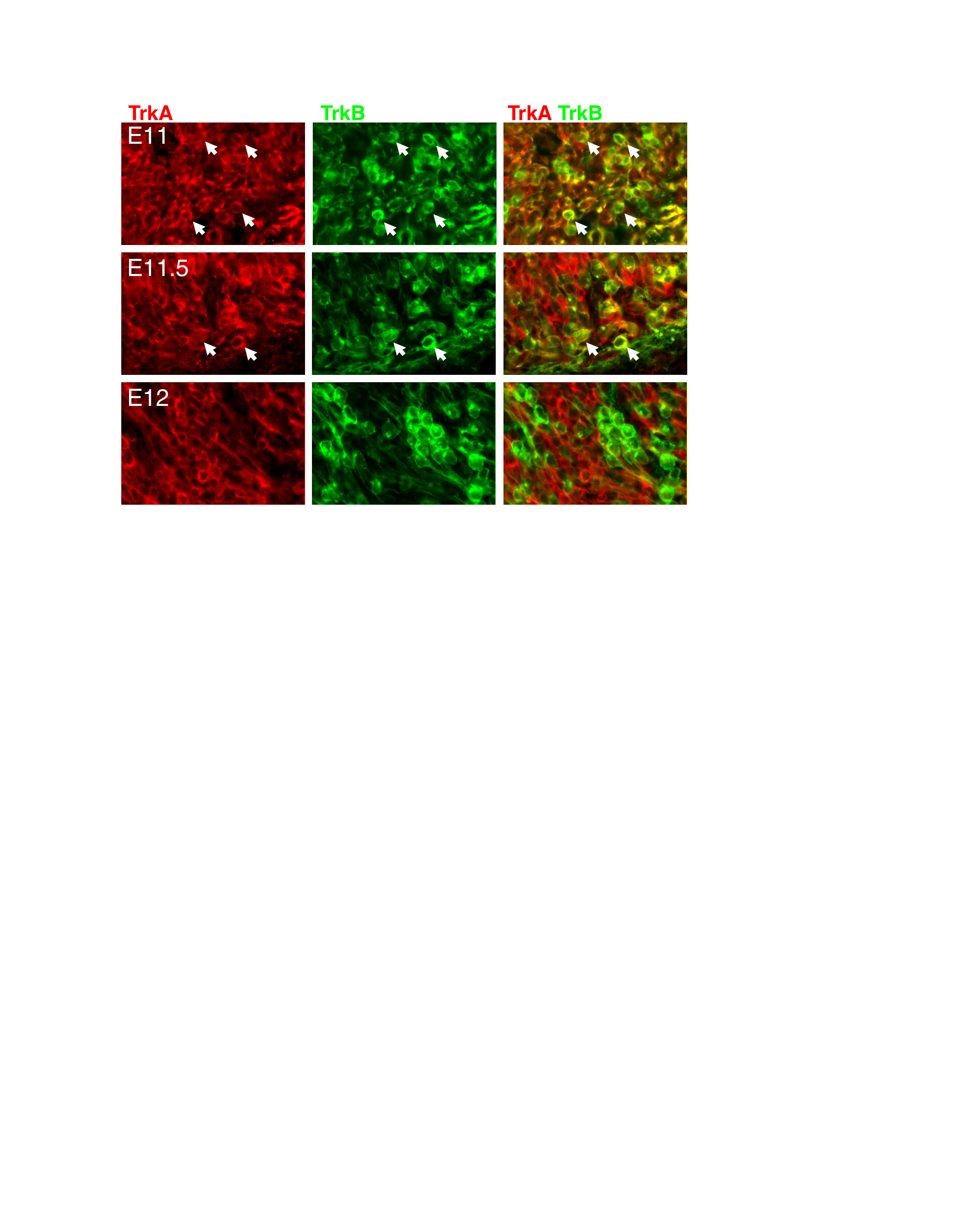

Supplement: Additional file 5 — Figure S2: Developmental segregation of TrkA and TrkB expression. The TG of control embryos were examined at E11, E11.5, and E12.0 in the horizontal plane. TrkA/B immunoreactive cells could no longer be detected at E12. Arrows indicate position of representative TrkA/B co-expressing cells. [file 1749-8104-5-3-S5.JPEG]

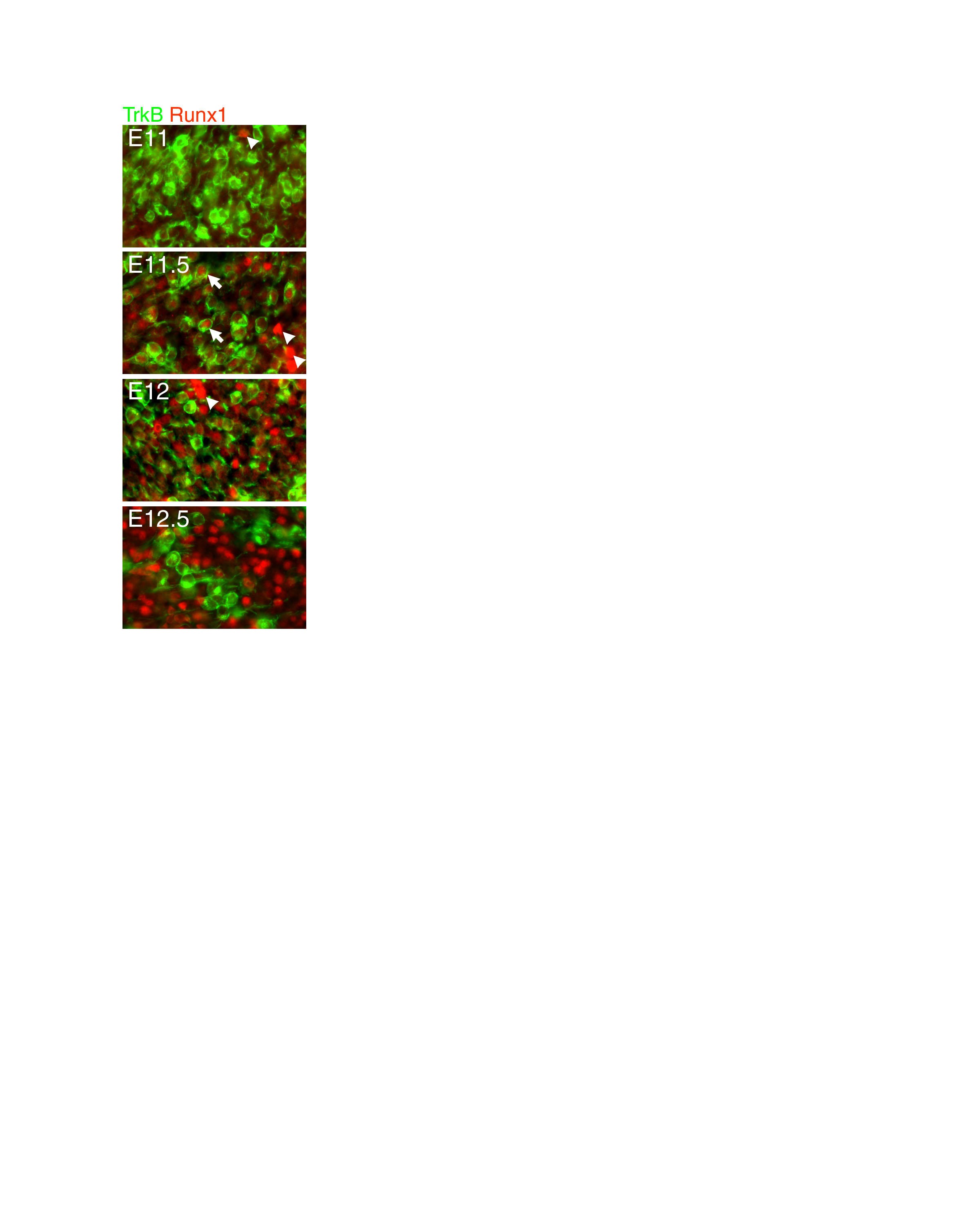

Supplement: Additional file 6 — Figure S3: Onset of Runx1 expression in the TG. The TG of control embryos were examined at E11, E11.5, E12 and E12.5 in the horizontal plane. Runx1 immunoreactivity was first detected at E11.5. At this stage occasional cells were observed which co-expressed Runx1 and TrkB (arrows), but co-expression was transient. Very bright cells indicated by arrowheads are blood/vascular artifacts. [file 1749-8104-5-3-S6.JPEG]
